# The role of mitochondrial DNA and RNA in promoting liver disease pathogenesis

**DOI:** 10.3389/fcell.2026.1855694

**Published:** 2026-09-28

**Authors:** Sanjeevani Raj, Anjali Warhade, Nikkee Kumari, Shweta Pandey, Mrigya Babuta

**Affiliations:** 1 Department of Animal Biology, School of Life Sciences, University of Hyderabad, Hyderabad, Telangana, India; 2 Government Vishwanath Yadav Tamaskar Postgraduate Autonomous College, Durg, Chhattisgarh, India

**Keywords:** Mitobiogenesis, DAMP, mt-dsRNA, ox-mtDNA, MDV, megamitochondria, MQC, DNA-sensing receptors

## Abstract

Mitochondria are a semi-autonomous organelle due to their unique structural organization and independent genetic system, and they play diverse roles in maintaining cellular homeostasis. The liver is the central metabolic organ and performs myriad functions, including energy production, metabolism, and detoxification. Liver diseases account for approximately two million mortalities per year. Mitochondrial dysfunction and damage are implicated in promoting the pathogenesis of different liver diseases. Mitochondria and mitochondrial nucleic acids, namely, mitochondrial DNA (mtDNA) and mitochondrial RNA (mtRNA), have emerged as regulators of innate immunity. Here, we first review the structural organization of mitochondria and the molecular mechanisms governing mitochondrial biogenesis, replication, and transcription. Next, we describe the mechanistic details by which mitochondrial dysfunction causes the release of mtDNA and mtRNA into the cytosol and extracellular milieu. Some of the mechanisms are through the opening of mitochondrial permeability transition pore (mPTP), voltage-dependent anion channel (VDAC) oligomerization, prohibitin (PHB1/2), mitochondria-derived vesicles (MDV), dysfunction of the mtRNA degradosomes, and senescence-associated secretory phenotype (SASP). Cell death pathways such as necrosis, necroptosis, and pyroptosis, in addition to perpetuating inflammation, also release mtDNA and mtRNA into the extracellular milieu. The released mtDNA and mtRNA act as damage associated molecular patterns (DAMPs) that engage various cytosolic DNA and RNA sensors and pattern recognition receptors (PRRs) such as cyclic guanosine-monophosphate-adenosine-monophosphate synthase (cGAS), toll-like receptor 9 (TLR9), toll-like receptor 3 (TLR3), Z-DNA-binding protein 1 (ZBP1), NOD-like receptor protein 3 (NLRP3), absent in melanoma 2 (AIM2), retinoic acid-inducible gene I (RIG-I), melanoma differentiation-associated protein 5 (MDA5), protein kinase R (PKR) and activate different signaling cascades downstream. This review provides a contemporary, comprehensive analysis of the available literature on the roles of mtDNA and mtRNA in liver diseases, including metabolic dysfunction-associated steatotic/steatohepatitis (MASLD/MASH), alcohol-associated liver disease (ALD), drug-induced liver injury (DILI), viral hepatitis, and hepatocellular carcinoma (HCC). Thus, the review highlights the potential of both mtDNA and mtRNA to serve as biomarkers of the pathological conditions of the liver, and the way the mitigation of mitochondrial dysfunction and the release of mtDNA or mtRNA can serve as potential therapeutic targets.

## 1 Introduction

The liver is a central integrative organ that supports immunity, detoxification and metabolism in the body (Trefts et al., 2017). It is composed of parenchymal and non-parenchymal cells formed from embryonic endoderm and mesoderm, respectively (Trefts et al., 2017; Kalra et al., 2026). Parenchyma of the liver is mainly constituted by hepatocytes, which are of epithelial origin and perform the majority of functions ascribed to the liver (Trefts et al., 2017; Kalra et al., 2026). Cholangiocytes are the second major cellular component of parenchymal cells, lining the bile duct lumen. The non-parenchymal cells include hepatic stellate cells (HSCs) of mesenchymal origin, which remain in a quiescent state in an uninjured liver and store most of the vitamin A (Trefts et al., 2017; Kalra et al., 2026; Yasaka et al., 2026). The liver also harbors resident macrophages known as Kupffer cells (KCs) which patrol the portal circulation to clear pathogens and engulf foreign particles, adopting pro- or anti-inflammatory phenotypes depending on the microenvironmental cues (Trefts et al., 2017; Kalra et al., 2026). Lastly, the specialized endothelial cells of the liver, known as liver endothelial sinusoidal cells (LSECs), possess the unique property to form fenestrated sieve plates that allow the exchange of proteins and nutrients from the bloodstream to various other liver cells (Trefts et al., 2017; Kalra et al., 2026). All these cells coordinately form the functional unit of the liver - the hepatic lobule.

The hexagonal hepatic lobule consists of the portal vein, hepatic artery, and bile duct, often referred to as the portal triad (Trefts et al., 2017; Kalra et al., 2026). These hepatic lobules are divided into three distinct metabolic zones with different oxygen levels, cell populations, and functions (Trefts et al., 2017; Kalra et al., 2026). Zone 1, the periportal region, receives most of the oxygenated blood and nutrients, and has a homogeneous population of hepatocytes, which are the primary site for oxidative metabolic processes such as beta-oxidation, gluconeogenesis, and bile formation, as well as support sinusoidal structure and angiogenesis via specialized HSCs and LSEC cells (Kalra et al., 2026; Ben-Moshe et al., 2019). Zone 2 serves as a buffer or transition zone. Zone 3, the pericentral region, receives the least oxygenated blood and hepatocytes in this zone are predominantly responsible for xenobiotic detoxification, biotransformation of drugs, synthesis of ketone bodies, glucose conversion into pyruvate, and fat synthesis (Trefts et al., 2017; Kalra et al., 2026; Yasaka et al., 2026; Ben-Moshe et al., 2019; Paris and Henderson, 2022; Manco and Itzkovitz, 2021). This zone is also enriched in HSCs, which regulate drug metabolism and promote liver regeneration, whereas LSECs in zones two and three are involved in innate immune activation (Ben-Moshe et al., 2019; Paris and Henderson, 2022; Manco and Itzkovitz, 2021). Liver zonation creates distinct metabolic environments across the lobule that define the abundance and function of the mitochondria, as well as their susceptibility to injury in different cell populations (Ben-Moshe et al., 2019; Paris and Henderson, 2022; Manco and Itzkovitz, 2021).

Mitochondria, widely recognized as the ‘powerhouse of the cell’, has an origin of an alpha-proteobacterium through endosymbiosis (Hess, 1965; Yapryntseva et al., 2024). It has various conserved characteristic features of its bacterial ancestors, such as two different membranes, i.e., inner mitochondrial membrane (IMM) and outer mitochondrial membrane (OMM) and circular double-stranded DNA with hypomethylated CpG motifs (Yapryntseva et al., 2024). IMM and OMM differ markedly in composition and structure where OMM resembles the eukaryotic membrane and IMM resembles bacterial membrane, with the presence of cristae. Ion gradient across the IMM drives ATP synthesis via oxidative phosphorylation (OXPHOS) which also serves as the intracellular source of reactive oxygen species (ROS) (Hess, 1965; Taft, 1964; Lardy and Ferguson, 1969). Under physiological conditions, ROS are produced by several enzymes, such as NADPH oxidases (NOXs), xanthine oxidase, and nitric oxide synthases functioning as a critical secondary messenger in numerous intracellular signaling pathways such as calcium signaling, modulating adaptive and protective responses under stress conditions (Zhang B. et al., 2022). Increasing intracellular calcium creates a driving force for mitochondrial calcium sequestration, mediated by the mitochondrial calcium uniporter (MCU) located in the IMM (Trefts et al., 2017; Hess, 1965; Alevriadou et al., 2021). This calcium influx in mitochondria stimulates pyruvate dehydrogenases, isocitrate dehydrogenases, and α-ketoglutarate dehydrogenases, thereby accelerating the tricarboxylic acid cycle, boosting ATP production, and potentially increasing the risk of electron leakage and ROS generation (Trefts et al., 2017; Hess, 1965; Lardy and Ferguson, 1969). Thus, beyond the classical role as “the powerhouse of the cell”, mitochondria are highly dynamic organelles structurally as well as at the genomic level, and are pivotal in maintaining cellular homeostasis and survival (Trefts et al., 2017; Hess, 1965; Gustafsson et al., 2016).

In this review we will discuss the biogenesis, release and role of mitochondrial DNA and RNA in the pathogenesis of liver disease.

## 2 Mitochondrial DNA and RNA - Biogenesis, structure, and function

Mitochondria are semi-autonomous organelles due to their unique structural organization and independent genetic system (Hess, 1965; Taft, 1964; Gustafsson et al., 2016). Mitochondria increase in size and number in response to environmental cues by a process called as mitobiogenesis (Popov, 2020). A coordinated interaction between mitochondrial and nuclear genes and the replication, transcription and translation of mitochondrial DNA (mtDNA) is necessary for mitobiogenesis (Popov, 2020). Interestingly mtDNA has an entirely distinct structure compared to nuclear DNA (nDNA) which is evident by the lack of cell cycle-dependent replication and its packaging with proteins other than histones, into compact units known as nucleoids (Gustafsson et al., 2016; Sharma and Sampath, 2019). mtDNA is composed of 16.5 kb closed-circular dsDNA having two strands: heavy (H) and light (L) strands, which consists of 16,569 bp encoding for 37 genes and 650 bp of non-coding region (Gustafsson et al., 2016; Cantatore and Attardi, 1980). These genes encode for 22 tRNAs, 2 rRNA (12S and 16S), and 13 polypeptide coding genes for OXPHOS (Gustafsson et al., 2016; Cantatore and Attardi, 1980).

mtDNA replication and transcription are highly interlinked processes, in which mitochondrial RNA polymerase (POLRMT) generates primers at heavy-strand and light-strand origins (Table 1) (Cantatore and Attardi, 1980; Park et al., 2023). The key enzyme involved in the replication is DNA polymerase gamma (POLγ), which exists as a heterotrimeric complex, its catalytic POLγA subunit has polymerization activity along with 3′-5′ exonuclease proofreading activity whereas POLγB accessory subunits help in enhancing the processivity and efficiency of replication (Park et al., 2023; Hecht, 1975). Alongside POLγ, TWINKLE helicase functions to facilitate efficient DNA synthesis by unwinding DNA in 5′-3′ direction (Korhonen et al., 2003). Mitochondrial single-stranded DNA-binding protein (mtSSB) stabilizes the long unwound single-stranded (ss) stretches of DNA at the replication fork (Jiang et al., 2021).

**TABLE 1:** List of proteins involved in mtDNA replication, transcription and maintenance.

​	Enzyme name	Full form	Functional role	References
Replication machinery	POLγA	Polymerase gamma A	Catalytic subunit of mtDNA polymerase	(Park et al., 2023; Falkenberg et al., 2024)
	POLγB	Polymerase gamma B	Accessory subunit of mtDNA polymerase	(Park et al., 2023; Falkenberg et al., 2024)
	TWINKLE	​	Replicative helicase	(Korhonen et al., 2003)
	mtSSB	Mitochondrial single-stranded DNA binding protein	It stabilizes the single-stranded DNA stretches at the replication fork	(Jiang et al., 2021; Miralles et al., 2014)
	PrimPol	Primase-Polymerase	Secondary primase/polymerase	(Torregrosa-Muñumer et al., 2017)
	RNase H1	​	RNA primer removal	(Ruhanen et al., 2011)
	MGME1	Mitochondrial genome maintenance exonuclease 1	DNA primer processing exonuclease	(Urrutia et al., 2022)
	DNA ligase III	​	Strand ligation	(Ruhanen et al., 2011)
	TOP1MT	Mitochondrial topoisomerase 1	Mitochondrial topoisomerase I	(Bhattacharjee and Das, 2025)
	TOP2α and TOP2β	Topoisomerase 2 alpha and topoisomerase 2 beta	Type IIA topoisomerases	(Bhattacharjee and Das, 2025)
Transcription machinery	POLRMT	Mitochondrial RNA polymerase	Core transcription enzyme	(Cantatore and Attardi, 1980; Park et al., 2023)
	TFAM	Mitochondrial transcription factor A	Promoter binding, DNA packaging	(Alam et al., 2003; Kaufman et al., 2007; Scarpulla et al., 2012)
	TFB2M	Mitochondrial transcription factor B2	Transcription initiation	(Ramachandran et al., 2017; Shokolenko and Alexeyev, 2017)
	TFB1M	Mitochondrial transcription factor B1	12S rRNA methylation	(Ramachandran et al., 2017; Shokolenko and Alexeyev, 2017; Sharoyko et al., 2014)
	TEFM	Mitochondrial transcription elongation factor	Transcription elongation	(Lei et al., 2024)
MTERF family	MTERF 1	Mitochondrial transcription termination factor 1	Prevents antisense transcription of rRNA genes	(Shokolenko and Alexeyev, 2017; Terzioglu et al., 2013; He et al., 2026)
	MTERF 2	Mitochondrial transcription termination factor 2	Nucleoid associated	(Shokolenko and Alexeyev, 2017; He et al., 2026; Han et al., 2016)
	MTERF 3	Mitochondrial transcription termination factor 3	Negative regulator of transcription	(Shokolenko and Alexeyev, 2017; He et al., 2026; Park et al., 2007)
	MTERF 4	Mitochondrial transcription termination factor 4	Forms complex with NSUN4; ribosome assembly	(Shokolenko and Alexeyev, 2017; He et al., 2026; Yakubovskaya et al., 2012; Cámara et al., 2011)
	NSUN 4	NOP2/Sun RNA methyltransferases	Methyltransferase, partners with MTERF4	(Yakubovskaya et al., 2012; Cámara et al., 2011)
RNA processing	MRPP1, MRPP2, MRPP3	Mitochondrial RNase P Protein	Subunits of the all-protein RNase P (5′ tRNA processing)	(Reinhard et al., 2017; Bhatta et al., 2021)
	ELAC2 (RNase Z)	ElaC ribonuclease Z	3′ tRNA processing	(Bhatta et al., 2025; Xue et al., 2024)

Current understanding of mitochondrial genome duplication is described by three distinct mechanistic models, and the first and to date, widely accepted model, is the strand-displacement model (Miralles et al., 2014). According to this model, replication of both strands occurs continuously without the formation of Okazaki fragments (Miralles et al., 2014). This model suggests that the replication begins at the heavy strand origin where TWINKLE helicase unwinds the double-stranded (ds) DNA ahead of POLγ, thus generating a long stretch of ss parental DNA, stabilized by mtSSB until the initiation of light strand replication (Jiang et al., 2021). The second model is the RNA incorporation throughout the lagging strand (RITOLS) model of replication, that relies on RNA to serve as the lagging strand template and is regulated by the balance between TWINKLE helicase activity and RNA transcript levels. Increased expression levels of TWINKLE helicase accelerates DNA unwinding and reduces the RNA incorporation (Korhonen et al., 2003). However, when RNA transcript levels, particularly of 7S RNA are abundant, it overrides the expression of TWINKLE, thus ensuring the presence of sufficient RNA molecules are available to coat the lagging strand, to protect it from nucleases and prevent aberrant RNA-DNA hybrid secondary structure (Korhonen et al., 2003; Pohjoismäki et al., 2010). During this process, the mtSSB proteins that coat the ss displaced parental strand are replaced by the RNA transcripts and form RNA-DNA hybrids that stabilize the displaced strands (Miralles et al., 2014). These hybrids are subsequently replaced by DNA as replication proceeds, yielding daughter mtDNA (Pohjoismäki et al., 2010). The strand-coupled model hypothesizes that the heavy and light strands are synthesized simultaneously, in a synchronous, bidirectional manner, predominantly producing double-stranded replication intermediates (Miralles et al., 2014; Cluett et al., 2018).

Mitobiogenesis is triggered by various physiological as well as pathological stimuli, which include increased energy demand, oxidative stress, hypoxia, nutrient deprivation, and mitochondrial damage, primarily through the activation of peroxisome proliferator-activated receptor coactivators 1 (PGC-1)-mediated signaling pathway (Popov, 2020; Liang and Ward, 2006). The family of PGC-1 cofactor proteins includes PGC-1α, PGC-1β and PGC-1γ (Liang and Ward, 2006). PGC-1α is the key regulator that initiates the pathway, either by phosphorylation or deacetylation, depending on the AMP/ATP ratio and acetyl-CoA levels (Liang and Ward, 2006). The activation of PGC-1α triggers a series of transcription factors (TFs), including nuclear respiratory factor 1 (NRF1), nuclear respiratory factor 2 (NRF2), and estrogen-related receptor α (ERRα) (Liang and Ward, 2006; Scarpulla et al., 2012). The final effector molecule of this signaling cascade is the mitochondrial transcription factor A (TFAM), which initiates the mtDNA replication and transcription (Liang and Ward, 2006; Scarpulla et al., 2012).

TFAM structurally associates with mtDNA and helps in the organization of mitochondrial nucleoids by bending, wrapping, and unwinding the mtDNA with the help of its two high mobility group (HMG)-box domains known as HMG box 1 and HMG box 2 (Rubio-Cosials et al., 2018; Hillen et al., 2017; Ramachandran et al., 2017; Shokolenko and Alexeyev, 2017). TFAM binds to the specific upstream sequence of light strand promoter (LSP) and heavy strand promoter (HSP) and the HMG-boxes clamp the mtDNA and introduces a U-turn of 180°, which causes the distortion in the B-form of mtDNA (Rubio-Cosials et al., 2018; Hillen et al., 2017; Ramachandran et al., 2017; Shokolenko and Alexeyev, 2017). This U-turn physically brings the upstream TFAM-binding site into close contact with the transcription start site (TSS), thus positioning TFAM’s C-terminal tail directly in contact with mitochondrial transcription factor B2 (TFB2M) and POLRMT, forming an initiation assembly (Table 1) (Rubio-Cosials et al., 2018; Hillen et al., 2017; Ramachandran et al., 2017; Shokolenko and Alexeyev, 2017). Close contact between TFB2M and POLRMT converts the closed promoter complex into an open complex which leads to the local unwinding of DNA at the TSS (Ramachandran et al., 2017; Shokolenko and Alexeyev, 2017). TFB2M stabilizes the unwound strand and POLRMT synthesizes RNA *de novo* in 5′ to 3′ direction, complementary to the template strand (Ramachandran et al., 2017; Shokolenko and Alexeyev, 2017). Once a short strand of 8–9 nucleotides of RNA is synthesized, POLRMT leaves the promoter, releasing TFAM/TFB2M complex and transitions into a stable, processive elongation complex (Ramachandran et al., 2017; Shokolenko and Alexeyev, 2017). POLRMT moves along the circular genome transcribing continuously as there are no intergenic gaps or terminator sequences between most genes (Ramachandran et al., 2017; Shokolenko and Alexeyev, 2017; Falkenberg et al., 2024). This generates a single polycistronic precursor transcript spanning nearly the entire H-strand or the entire L-strand (Shokolenko and Alexeyev, 2017; Falkenberg et al., 2024). This newly synthesized mtRNA becomes organized into discrete ribonucleoprotein complexes known as mtRNA granules (MRGs), which typically localize adjacent to mitochondrial nucleoids (Antonicka and Shoubridge, 2015). Since the transcripts produced from H-strand (sense) and L-strand (antisense) share extensive sequence complementarity across the genome, co-localization of these complementary single-stranded transcripts within the mitochondrial matrix allows Watson-Crick base pairing, yielding stable dsRNA duplexes (Shokolenko and Alexeyev, 2017; Falkenberg et al., 2024). Under physiological conditions, mt-dsRNA is a low-level byproduct that is predominantly restricted within the matrix by degradosome components suppressor of Var1 3 (SUV3), which prevents the accumulation of mt-dsRNA and polynucleotide phosphorylase (PNPase) that is responsible for the degradation of accumulated mt-dsRNA (Shokolenko and Alexeyev, 2017; Pajak et al., 2019; Santonoceto et al., 2024). However, a small fraction of mt-dsRNA leaks into the cytosol, where it contributes to baseline activation of cytosolic nucleic acid sensors Retinoic acid-inducible gene I (RIG-I) and melanoma differentiation-associated protein 5 (MDA5) (Dhir et al., 2018). During the termination process at the heavy strand, mitochondrial transcription termination factor 1 (MTERF1) binds within the tRNA-Leu (UUR) gene of 16S rRNA and causes POLRMT to end (Shokolenko and Alexeyev, 2017; Terzioglu et al., 2013). The released transcript is excised by RNase P at 5′ end and RNase Z/ELAC2 at the 3′ end, thus releasing the flanking mRNAs and rRNAs (Bhatta et al., 2025; Xue et al., 2024). Furthermore, processing of these RNA transcripts occurs co-transcriptionally or immediately post-transcriptionally at the nuclear periphery where the mRNAs are polyadenylated by mitochondrial poly(A) polymerase (mtPAP), rRNAs are methylated and assembled into mitoribosomal subunits, and tRNAs undergo base modification and CCA addition (Shokolenko and Alexeyev, 2017; Nagaike et al., 2005). After the processing of these transcripts, their translated products are correctly localized and assembled, however since individual nucleoids typically harbor only one to two copies of mtDNA and the resulting mtRNA-derived products are therefore unevenly distributed across the mitochondrial population (Shokolenko and Alexeyev, 2017; Nagaike et al., 2005). To offset this inherent heterogeneity, mitochondria undergo fusion, allowing intact genomes and functional transcripts to be shared across the network.

Mitochondria fusion is a dynamic process that merges the mitochondrial network to redistribute the genomic and transcript content (Twig et al., 2008). The fusion occurs between the OMM and IMM of the newly synthesized and the existing mitochondria (Twig et al., 2008). The fusion of OMM is aided by two residential large membrane GTPases, Mitofusin 1 (MFN1) and Mitofusin 2 (MFN2) (de Brito and Scorrano, 2008; Qi et al., 2016; Zacharioudakis et al., 2022). MFN2, also localized to endoplasmic reticulum (ER) membrane, facilitates coupling between ER and mitochondria, thereby promoting mitochondrial calcium uptake (de Brito and Scorrano, 2008; Qi et al., 2016; Zacharioudakis et al., 2022). IMM fusion is orchestrated by optic atrophy protein (OPA1), a dynamin-related GTPase, together with cardiolipin (Ge et al., 2020). Various studies show that a combination of different OPA1 isoforms is needed for the IMM fusion, however, the detailed mechanism is not fully known (Ge et al., 2020; Song et al., 2007). Mitochondrial fusion also aids in the maintenance of mitochondrial integrity and functional complementation (Twig et al., 2008; Zacharioudakis et al., 2022; Sabouny and Shutt, 2020). However, an imbalance in mitochondrial fusion dynamics can promote the formation of megamitochondria by facilitating the continuous merging of individual mitochondria into large, interconnected structures with pale matrices (Twig et al., 2008; Wakabayashi, 2002). Megamitochondria are commonly observed in certain liver diseases, which will be discussed in the respective liver etiologies in the upcoming section. Another coordinated process that helps in cellular homeostasis by maintaining mitochondrial quality and distribution is mitochondrial fission (Twig et al., 2008; Sabouny and Shutt, 2020). This predominantly occurs at the contact sites between the ER and mitochondria (Sabouny and Shutt, 2020). A key mediator of this process is dynamin-related protein (DRP1), a cytosolic GTPase whose activation depends on phosphorylation by different kinases at various serine residues (Sabouny and Shutt, 2020; Smirnova et al., 2001). Upon receiving certain cues and with the help of outer membrane receptor proteins like mitochondrial fission factor (MFF), mitochondrial dynamics protein (MiD49), MiD51, and mitochondrial fission one protein (FIS1), DRP1 is translocated onto the OMM and forms ring-like oligomeric structures, which constrict the mitochondria and result in its division (Losón et al., 2013).

Disruption of either mitochondrial fusion or fission processes by factors such as excessive ROS production, replication errors, and a limited number of DNA repair enzymes can impair mitochondrial function, thereby promoting apoptosis and cellular damage (Twig et al., 2008; Sabouny and Shutt, 2020). However, this disruption of the fusion-fission balance is not always pathological; under mild or transient stress, cells mount an adaptive response known as stress-induced mitochondrial hyperfusion (SIMH) (Twig et al., 2008; Das and Chakrabarti, 2020). SIMH is MFN1-driven response and is regulated by metalloprotease overlapping activity with m-AAA protease (OMA1) (Das and Chakrabarti, 2020). OMA1 regulates SIMH by cleaving OPA1 (Das and Chakrabarti, 2020). When OMA1 is suppressed, sustained OPA1-mediated fusion occurs, which elongates the mitochondrial network, maximizing ATP output, and thereby protecting mitochondria from mitophagy (Das and Chakrabarti, 2020).

During chronic stress, cells use a mitochondrial quality control (MQC) system that maintains mitochondrial integrity and balance between fusion and fission (Twig et al., 2008). MQC selectively removes damaged mitochondria via mitophagy and directly repairs oxidative DNA damage through the mitochondrial base excision repair (BER) pathway (Copeland and Longley, 2008; Jun et al., 2022). BER repairs the oxidative and alkylation-mediated DNA damage through DNA glycosylases, apurinic endonuclease 1 (APE1), flap endonuclease 1 (FEN1), DNA ligase III and DNA polymerase γ (POLγ) (Ranalli et al., 2002). The BER pathway utilizes glycosylases like 8-oxoguanine DNA glycosylase 1 (OGG1) to detect and remove 8-oxodeoxyguanine (8-oxo-dG), which is highly mutagenic, followed by cleavage of the phosphodiester backbone by APE1 (Mullins et al., 2019). Ultimately, DNA polymerase fills the remaining gap, and DNA ligase III seals the nick (Jun et al., 2022). MQC is also directly linked to mtRNA transcription at two levels; first as an adaptive QC response to increase mitochondrial function, PGC-1α-NRF1/2-TFAM signaling is activated which initiates mtDNA transcription and thereby increase mtRNA output and mitochondrial respiratory capacity (Liang and Ward, 2006; Scarpulla et al., 2012; Wu et al., 1999; Gleyzer et al., 2005). Second, mitochondrial degradosome act as QC degradation machinery and prevent the accumulation of dsRNA/G-quadruplex-forming RNA (Pajak et al., 2019; Santonoceto et al., 2024; Pietras et al., 2018). Failure of this surveillance system causes dsRNA/mtRNA accumulation and leakage (Pietras et al., 2018).

Thus, the coordinated balance between mitochondrial fission and fusion is essential for preserving mitochondrial integrity, quality control, and cellular homeostasis, thereby directly influencing the overall mitochondrial functionality.

Mitochondria play key physiological roles, primarily involving energy metabolism, signaling, and survival and serve as the primary site of ATP production via OXPHOS (Table 2) (Hess, 1965; Lardy and Ferguson, 1969). OXPHOS is driven by electrons from dehydrogenases flowing sequentially through complexes I-IV of the respiratory chain, with complexes I, III, and IV pumping protons across IMM to build proton motive force (PMF) that complex V then harnesses to phosphorylate ADP into ATP. Mitochondria also act as oxygen sensors; for instance, in vascular smooth muscle cells (SMCs), complex IV acts as the sensory antenna (Pak et al., 2022). Oxygen binds to the ferrous iron of heme a3 in the binuclear center of complex IV (Pak et al., 2022). During acute hypoxia, limited O_2_ availability at the heme a3-CuB binuclear center impairs its function as the terminal electron acceptor, slowing the reduction of oxygen to water and causing upstream electron backpressure that reduces overall, ETC flux, causing ROS overproduction through complex III (Pak et al., 2022). ROS, in turn, inhibits K^+^ channels, depolarize the membrane, and lead to calcium influx into SMCs, driving pulmonary vasoconstriction that matches blood flow to tissue oxygen demand (Pak et al., 2022). OXPHOS is metabolically coupled to the TCA cycle and β-oxidation, which supply the NADH and FADH_2_ donating electrons into the ETC (Hess, 1965; Lardy and Ferguson, 1969; Van Waveren et al., 2006). Excessive electron flux, however, drives electron leakage at complexes I and III, generating superoxide and peroxide ions even under physiological conditions (Hess, 1965; Mitchell, 1961; Guo et al., 2018). While moderate ROS supports signaling and redox regulation, excess ROS causes oxidative stress, mitochondrial dysfunction and development of various diseases (Lennicke and Cochemé, 2021).

**TABLE 2:** List of proteins encoded by mtDNA.

​	Protein name	Full form	Functional role	References
Complex I (NADH dehydrogenase)	ND1	NADH:Ubiquinone Oxidoreductase Core Subunit 1	Involved in ubiquinone binding and electron transfer from NADH to ubiquinone	(Anderson et al., 1981)
​	ND2	NADH:Ubiquinone Oxidoreductase Core Subunit 2	Participates in transmembrane proton translocation coupled to electron transfer	(Anderson et al., 1981)
​	ND3	NADH:Ubiquinone Oxidoreductase Core Subunit 3	Small core subunit; part of the interface between the matrix arm and membrane arm. Important for complex I assembly	(Chomyn et al., 1985)
​	ND4	NADH:Ubiquinone Oxidoreductase Core Subunit 4	Central component of proton-pumping machinery; interacts with ND1, ND2, and ND5 in the membrane arm	(Anderson et al., 1981)
​	ND4L	NADH:Ubiquinone Oxidoreductase Core Subunit 4L	Small accessory subunit. Stabilizes ND4 and participates in complex I assembly	(Chomyn et al., 1985)
​	ND5	NADH:Ubiquinone Oxidoreductase Core Subunit 5	Largest mtDNA encoded complex I subunit. Critical for proton translocation	(Chomyn et al., 1985)
​	ND6	NADH:Ubiquinone Oxidoreductase Core Subunit 6	Membrane-arm core subunit involved in proton translocation and stabilization of complex I also contributes to proper assembly of the distal membrane arm	(Chomyn et al., 1986)
Complex III (Cytochrome bc1 complex)	Cytochrome b*	​	Central transmembrane subunit of complex III that contains the Q_o_ and Q_i_ quinone-binding sites and mediates electron transfer from ubiquinol to cytochrome c via the Q-cycle	(Anderson et al., 1981)
Cytochrome IV (cytochrome c oxidase)	COI/COXI	Cytochrome c Oxidase Subunit I	Catalytic core subunit of cytochrome c oxidase containing the heme a and heme a_3_-CuB catalytic center, where molecular oxygen is reduced to water	(Anderson et al., 1981)
​	COII/COXII	Cytochrome c Oxidase Subunit II	Accepts electrons directly from cytochrome c and donates them to heme a of COXI.	(Anderson et al., 1981)
​	COXIII	Cytochrome c Oxidase Subunit III	Structural subunit that stabilizes the catalytic core and contributes to proton transfer efficiency and regulation of cytochrome c oxidase activity	(Anderson et al., 1981)
Complex V (ATP synthase)	ATP6	ATP Synthase Membrane Subunit a (Subunit 6)	Creates two aqueous half-channels through which protons translocate across the inner mitochondrial membrane	(Anderson et al., 1981)
​	ATP8	ATP Synthase Membrane Subunit 8 (Subunit A6L)	Anchors the a-subunit complex to the F_1_ domain to prevent futile rotation during proton-driven ATP synthesis	(Mariottini et al., 1983)

* Cytochrome b gene encodes a 187 aa protein, CYTB-187 A A, which is translated in the cytoplasm and interacts with the mitochondria phosphate carrier protein and helps in ATP, production (Hu et al., 2024).

Mitochondrial dysfunction with enhanced ROS production and increased calcium levels, leads to the release of mtDNA and mt-dsRNA into the cytosol.

## 3 Mechanism of mitochondrial DNA and RNA release

Mitochondria are transcribed bi-directionally from both sides of the strands, which makes mitochondria a source of two separate but mechanistically overlapping nucleic acid species-mtDNA and mt-dsRNA. When either of these molecules enters the cytosol, they elicit an immune response and are recognized as a damage-associated molecular patterns (DAMPs).

mtDNA is highly susceptible to damage caused by oxidative stress, formation of lipid peroxidation products, replication errors, and metabolic overload which causes formation of mtDNA lesions such as oxidized bases and single or double strand breaks (Sharma and Sampath, 2019; Hsu et al., 2013). In case of mtRNA, mitochondrial degradosome complex dysfunction because of excessive ROS and stress-induced hyper transcription causes accumulation of unstable mtRNA transcripts (Santonoceto et al., 2024). Accumulation of damaged mtDNA and mt-dsRNA followed by the failure in the quality control mechanism such as mitochondrial fusion, fission, mitochondrial degradosome complex and BER promotes their release from mitochondria to cytosol, as well as extracellular milieu (Dhir et al., 2018; Riley et al., 2018).

Upon mitochondrial dysfunction, mtDNA is released into the cytosol as well as into the extracellular space in different structural forms; it can either be a free DNA molecule comprising a full-length circular genome with or without bound with TFAM (Alam et al., 2003; Kaufman et al., 2007; Al Amir Dache and Thierry, 2023; Giordano et al., 2025; West and Shadel, 2017). Furthermore, mtDNA is also found as protein-bound fragments of DNA, oxidised, non-oxidised, linear single or ds forms, DNA-RNA hybrids, and either in B or Z conformations (Giordano et al., 2025; Wang and Xu, 2025; Newman et al., 2024). mtRNA can be released as both long antisense mt-dsRNA and short 5′-triphosphate containing transcripts which are generated at site of mtDNA damage (Yan et al., 2025). All these conformational forms are recognized by pattern recognition receptors (PRRs) upon their release into the cytosol (Giordano et al., 2025; Wang and Xu, 2025).

The release of mtDNA and mt-dsRNA is currently attributed to several overlapping mechanisms. These include leakage through the BAX/BAK-mediated mitochondrial outer membrane permeabilization (MOMP), voltage-dependent anion channel (VDAC) oligomerization, and gasdermin (GSDM)-mediated pore formation (Dhir et al., 2018; Giordano et al., 2025; Cosentino et al., 2022; Yamazaki and Galluzzi, 2022; Riley et al., 2018; Yasuda et al., 2026; Xian et al., 2022; Kim et al., 2019; Gogvadze and Zhivotovsky, 2025; Bernardi et al., 2023; Krieger et al., 2024; O’Carroll et al., 2024; Neel et al., 2023; Miao et al., 2023; de Torre-Minguela et al., 2021; Afaq et al., 2025). However, there are certain mechanisms which are studied in context of either mtDNA or mt-dsRNA which will also be elaborated in this section.

BAX/BAK-mediated MOMP pathway intersects at the critical step of intrinsic apoptosis, triggered by excessive ROS production or calcium imbalance (Cosentino et al., 2022; McArthur et al., 2018; Carvalho et al., 2004). In this process, pro-apoptotic proteins BAK and BAX oligomerize towards the OMM forming large pores which leads to its destabilization, causing the release of cytochrome c which activates apoptotic protease activating factor 1 (APAF1) and the caspase cascade (Dhir et al., 2018; Cosentino et al., 2022; McArthur et al., 2018; Carvalho et al., 2004). The structural distortion caused by pore formation allows mtDNA and mt-dsRNA to leak from the mitochondrial matrix into the cytosol (Dhir et al., 2018; Riley et al., 2018; Yasuda et al., 2026; Krieger et al., 2024). Recently, Yasuda et al., showed that mitochondrial hyperfusion due to DRP1 deficiency, causes accumulation and release of mt-dsRNA in cytosol via BAX/BAK pathway (Yasuda et al., 2026).

Under homeostatic condition VDAC1 acts as gatekeeper of the OMM, however under stress, it oligomerizes and allows the export of mtDNA and mt-dsRNA into the cytosol (Xian et al., 2022; Krieger et al., 2024; Camara et al., 2017). Interestingly mtDNA can orchestrate its own release by binding to the N-terminus of VDAC1, causing oligomerization and pore formation (Kim et al., 2019). In this mechanism, vesicles derived from the IMM (VDIMs) formed by VDAC1-mediated herniation of IMM, are packaged with oxidised mtDNA (ox-mtDNA) and mitochondrial nucleoids (Xian et al., 2022; Krieger et al., 2024; Camara et al., 2017). In case of mt-dsRNA, VDAC1/2 and inner membrane protein prohibitin (PHB1/2) plays a crucial role (Krieger et al., 2024). PHB1/2 forms a dynamic ring-like structure complex along with PNPase which is localized on either side of IMM (Krieger et al., 2024). This ring-like structure helps in the release of mt-dsRNA from matrix to inter-membrane space, which is exported out in the cytosol by VDAC1/2 (Krieger et al., 2024). Studies have shown that inhibiting either VDAC1/2 or PHB1/2 attenuates the export of mt-dsRNA to the cytosol (Krieger et al., 2024). Furthermore, metabolic stress induced due to itaconate-mediated inhibition of succinate dehydrogenase can also lead to VDAC1 dependent mt-dsRNA release in macrophages (O’Carroll et al., 2024). Recently, another mechanism of mt-dsRNA and mtDNA release, facilitated by gasdermin-dependent pore formation, has been reported (Neel et al., 2023; Miao et al., 2023; Afaq et al., 2025). GSDMD and GSDME, cleaves caspase-1 and caspase-3, which causes pore formation in the plasma membrane and mitochondria, leading to mitochondrial collapse, causing accumulation of mtDNA and mt-dsRNA in cytoplasm, and their subsequent escape into the extracellular space (Neel et al., 2023; Miao et al., 2023; de Torre-Minguela et al., 2021; Afaq et al., 2025).

Another mechanism associated with the release of mtDNA is via mitochondrial permeability transition pore (mPTP) opening. mPTP is a calcium and voltage-sensitive channel located in the IMM, which opens during calcium overload and oxidative stress (Bernardi et al., 2023; Xian et al., 2022). Sustained opening of mPTP disrupts mitochondrial membrane potential, inhibits respiration, increases ROS production, causes mitochondrial swelling followed by the rupturing of the IMM and trigger the release of mtDNA and pro-apoptotic proteins inside the cytosol (Bernardi et al., 2023; Xian et al., 2022). Impaired mitochondrial packaging caused by TFAM depletion, along with mtDNA instability, also contributes to mtDNA release through mPTP (Ding K. et al., 2025; Bryant et al., 2022). Additionally, the elevated level of cytosolic Ca^2+^ leads to its accumulation via MCU causing the mPTP opening (Cheng et al., 2026).

Besides ROS-mediated membrane permeabilization and mPTP opening, dysfunctional MQC is also crucial for the mtDNA release to the cytosol (Busch et al., 2014). For instance, dysfunctional mitophagy caused by the loss of PTEN-induced putative kinase 1 (PINK1) or Parkin, a regulator protein responsible for identifying degraded mitochondria and their removal is prevented, leading to increased cytosolic and circulating mtDNA levels (Kamat et al., 2014). Moreover, alterations in proteins regulating mitochondrial fusion and fission, such as DRP1, FIS1, MFN1, and MFN2, promote mtDNA fragmentation, destabilize nucleoids, as well as permit mtDNA leakage through endosomal pathways or directly into the cytosol (Losón et al., 2013; Wu et al., 2011).

Recently, a new mechanism of vesicle-mediated release of mtDNA has been identified. Studies show that the damaged mtDNA recruits endosomes near the mitochondrial regions, which contain mtDNA nucleoids (Prashar et al., 2024; Sen et al., 2023; Picca et al., 2023). Damaged mtDNA is sequestered in the Ras-associated binding 5 (RAB5)-positive early endosomes and later trafficked to the RAB7 late endosomes (Hammerling et al., 2017). Rupture of these late endosomes in conditions of mitochondrial stress, RAB7 deficiency releases their contents, including mtDNA, into the cytosol (Sen et al., 2023; Hammerling et al., 2017; Sen et al., 2022). The release of mtDNA is also facilitated by mitochondria-derived vesicles (MDVs), which are smaller than endosomes (Picca et al., 2023; Adla et al., 2026). While the exact mechanism by which mtDNA exits these vesicles remains unclear, studies suggest that increased levels of certain metabolic intermediates, such as fumarate, can influence mtDNA exit from MDVs (Picca et al., 2023; Zecchini et al., 2023). This process appears to involve a pathway that includes sorting nexin 9 (SNX9), vacuolar protein sorting-associated protein 35 (VPS35), and mitochondrial Rho GTPase (MIRO) (Sen et al., 2022; König et al., 2021). Recently Nguyen et al., showed that OMM protein mitochondrial anchored protein ligase (MAPL) promotes trafficking of MDV packaged with mtDNA to lysosomes, followed by gasdermin mediated pore formation in lysosomes and release of mtDNA in cytosol (Nguyen et al., 2025).

Different cell-death pathways, such as necrosis, necroptosis, and pyroptosis, contribute to the release of mtDNA (Giordano et al., 2025; Gogvadze and Zhivotovsky, 2025). For instance, in necroptosis when ligand binds with tumor necrosis factor receptor 1 (TNFR1), receptor-interacting protein kinase 1 (RIPK1) undergoes autophosphorylation which promotes binding of receptor-interacting protein kinase 3 (RIPK3) (Wang WY. et al., 2022; Ding Z. et al., 2025). RIPK3 then phosphorylates mixed lineage kinase domain-like pseudokinase (MLKL), which acts as an executioner and translocate to the plasma membrane, causing pore formation and swelling that facilitates mtDNA release (Wang WY. et al., 2022; Ding Z. et al., 2025). Some studies have shown that cytosolic mtDNA binds to ZBP1 and activates necroptosis via MLKL (Lei et al., 2023). Similarly, in ferroptosis, an iron-dependent form of cell death, the release of mtDNA into the extracellular milieu and the cytosol occurs (Wu et al., 2021; Zhou et al., 2026). In ferroptosis, Fe^2+^ causes accumulation of ROS which alters membrane potential and triggers the opening of mPTP, however, under physiological condition mPTP is in a closed state by mitochondrial matrix protein, cyclophilin D (Zhou et al., 2026).

Similarly, dysfunction of the mtRNA degradosome complex comprising the helicase SUV3 (SUPV3L1) and PNPase, which works with NOP2/Sun RNA methyltransferase 4 (NSUN4) and complement C1q-binding protein (C1QBP), lead to the mt-dsRNA release into the cytosol (Pajak et al., 2019; Dhir et al., 2018). NSUN4 causes m5C modifications onto mtRNAs which are recognized by C1QBP to recruit PNPase for RNA turnover (Dhir et al., 2018; Kim et al., 2024). Thus, deletion of SUV3 leads to the accumulation of mt-dsRNA which are released to the cytosol in absence of PNPase as well as loss of NSUN4–C1QBP–PNPT1 methylation axis also cues this mt-dsRNA release (Dhir et al., 2018; Kim et al., 2024). The deletion of C1QBP alone can also induce cytosolic efflux of mt-dsRNA after it has been modified by NSUN4, however lack of m5C modification on mtRNA due to NSUN4 depletion causes mtRNA to be retained inside the mitochondria showing not only the proteins but the chemical modification of mt-dsRNA is also necessary for its release (Kim et al., 2024). Another study used CRISPR-Cas9 screening of RNA-binding proteins (RBP) and found 89 RBPs to have the ability to cause mt-dsRNA accumulation when deleted or mutated (Kim et al., 2024).

The release of mt-dsRNA is found to be a characteristic feature of senescent cells known as senescence-associated secretory phenotype (SASP) regulated by MFN1, where inhibition of MFN1 protein reduces SASP (Victorelli et al., 2025; López-Polo et al., 2024). Recent studies have shown ionizing radiation damages mitochondria and induce the release of mt-dsRNA which causes activation of immune response after being detected by PRRs (Miao et al., 2023; Zhang A. et al., 2025). Furthermore, urolithin can attenuate damage caused by radiation by inducing mitophagy (Zhang A. et al., 2025).

Collectively, several pathways and cellular and metabolic conditions contribute to the release of mtDNA and mt-dsRNA. Once released, DNase, especially DNase I and DNase I-like 3, degrades circulating cell-free mtDNA (ccf-mtDNA) and mtDNA released from dying cells, thereby acting as a defense mechanism and preventing the activation of innate immune pathways (Giordano et al., 2025; West and Shadel, 2017; Trumpff et al., 2021; VanPortfliet et al., 2024). Similarly, the RNA degradosome proteins clears the mtRNA however, under sustained oxidative stress, loss of SUV3 and PNPT1 protein, clearance of both, the mtDNA and mt-dsRNA, becomes ineffective, leading to their accumulation in the cytoplasm and extracellular spaces, where it serves as mitochondrial damage-associated molecular patterns (mtDAMPs) (Pajak et al., 2019; Santonoceto et al., 2024; Yan et al., 2025).

## 4 Recognition of mitochondrial DNA and RNA as damage-associated molecular pattern (DAMP)

Released mtDNA and mt-dsRNA in the cytosol act as DAMPs which are recognized by different PRRs and activate several downstream immune responses. As mentioned in previous section released mtDNA can be found in various forms in the cytosol, such as fragments, intact nucleoids, and ox-mtDNA, which are detected by PRRs and sensor proteins, such as toll-like receptor 9 (TLR9), the NOD-like receptor protein 3 (NLRP3), absent in melanoma 2 (AIM2) inflammasome, and cyclic GMP–AMP synthase (cGAS) (Zanini et al., 2023; Bliksøen et al., 2016; Pan et al., 2018; Xu et al., 2021; White et al., 2014) (Figure 1). Followed by activation of downstream signaling pathways, including mitogen-activated protein kinases (MAPKs), nuclear kappa-light–chain-enhancer of activated B cells (NF-κB), and interferon regulatory factor (IRF), that further activate different pro-inflammatory cytokines, co-stimulatory molecules, and chemokines, as well as activation of innate immune effector cells (Ding Z. et al., 2025; Bliksøen et al., 2016; Xu et al., 2021; White et al., 2014).

**FIGURE 1:**
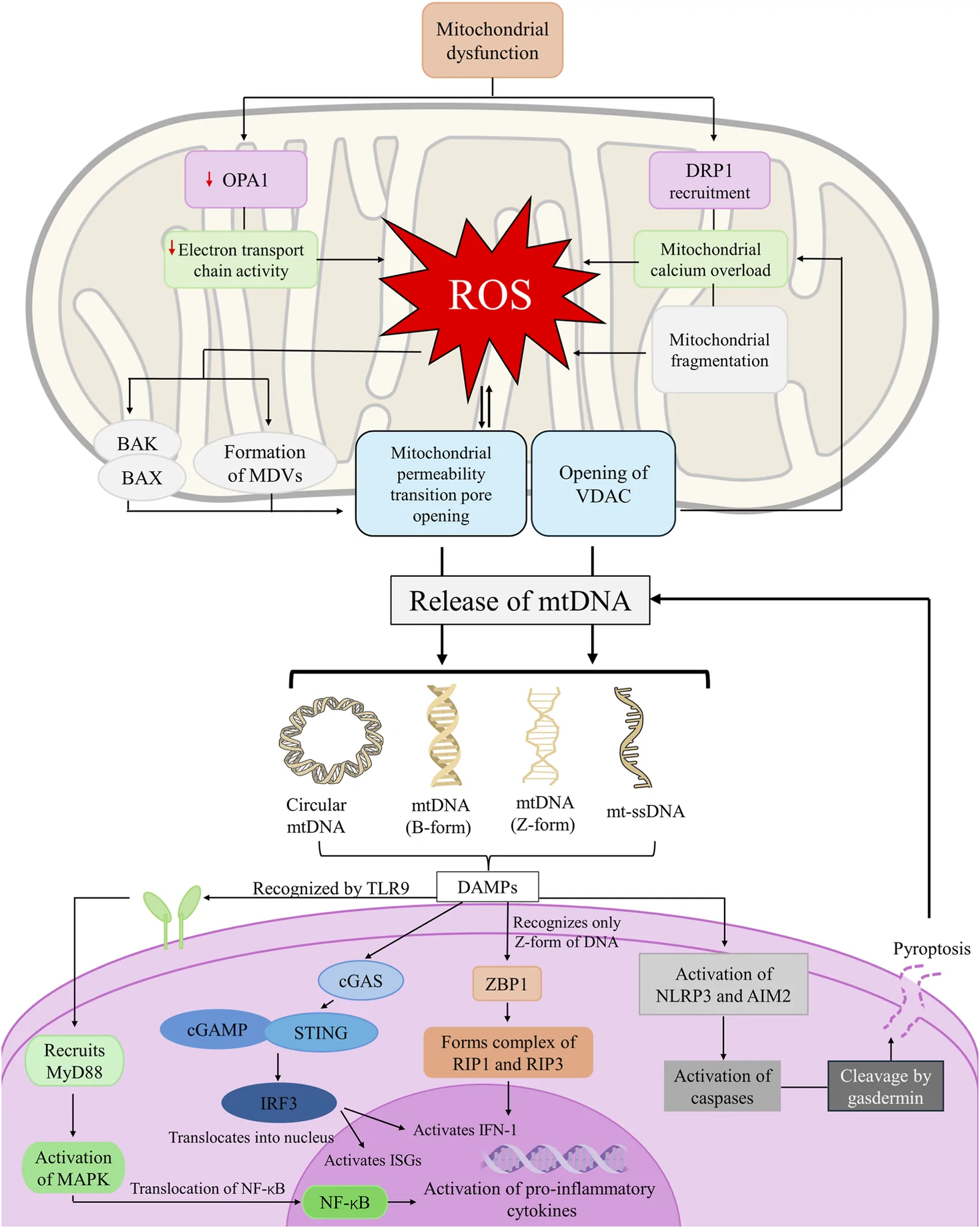
Schematics showing the mechanism of mtDNA release and activation of various downstream pathways. Liver disease–associated mitochondrial dysfunction promotes recruitment of DRP1 and downregulation of OPA1, leading to impaired mitochondrial dynamics. DRP1 recruitment induces mitochondrial calcium overload and mitochondrial fragmentation. Both processes contribute to excessive ROS generation. Elevated ROS promotes BAX/BAK complex formation and also stimulates the generation of MDVs. Together with ROS, these events facilitate the opening of the mPTP, further amplifying ROS production. Additionally, oligomerization of VDAC enhances its opening and contributes to mitochondrial calcium overload. Opening of VDAC and mPTP mediates the release of mtDNA in multiple forms, including B-form DNA, Z-form DNA, ss-mtDNA, and circular DNA, which acts as damage-associated molecular patterns (DAMPs). These mtDNA act as DAMPs. TLR9 recognizes mtDNA and activates the MyD88-dependent pathway, leading to MAPK and NF-κB. Cytosolic mtDNA is also sensed by cGAS-STING-IRF3, ZBP1-RIP1-RIP3, NLR3 and AIM2.

cGAS is a cytosolic sensor for dsDNA and produces 2′,3′-cyclic GMP–AMP (cGAMP) upon activation (Babuta et al., 2026; Margolis et al., 2017). The cytosolic mtDNA binds to cGAS and forms a cGAS-DNA complex, which catalyses the formation of cGAMP (Margolis et al., 2017; Lai et al., 2025). cGAMP binds to stimulator of interferon genes (STING) and activates TANK-binding kinase 1 (TBK1), which phosphorylates both STING and IRF3 (Babuta et al., 2026; Margolis et al., 2017). Phosphorylated IRF3 dimerizes and localizes to the nucleus, where it induces transcription of type I interferons (IFN-I) and interferon-stimulated genes (ISGs), which causes sterile inflammation and cellular senescence (Margolis et al., 2017; Lai et al., 2025). Moreover, STING activation simultaneously activates NF-κB by recruiting IκB kinase (IKK), which promotes the release of IL-6 and TNF-α (White et al., 2014; Lai et al., 2025). Moreover, interferon-γ-inducible protein 16 (IFI16), another cytosolic DNA sensor cooperates with cGAS to activate STING and detects partially degraded mtDNA in lysosomes and activates an IFN-I response (Almine et al., 2017; Matsui et al., 2021).

Z-DNA-binding protein 1 (ZBP1), recognizes only Z-form of mtDNA and not the B-form. It binds to and stabilizes Z-form of mtDNA, and forms a complex of cGAS, RIPK1, and RIPK3, and parallelly activate IFN-I signaling, which enhances inflammation (Lai et al., 2025; Song et al., 2025; Zheng and Kanneganti, 2020; Chen C. et al., 2025). This suggests that the innate immune sensors can distinguish between structurally distinct mtDNA species to elicit immune response (Song et al., 2025; Chen C. et al., 2025).

Inflammasomes such as NLRP3 and AIM2 detects ox-mtDNA and mtDNA (Xian et al., 2022; Xu et al., 2021; Lackner et al., 2025; Cabral et al., 2023). Interestingly, NLRP3 inflammasome has an opposing role, it can cleaves ox-mtDNA but is also activated by the ox-mtDNA (147,148). ox-mtDNA interacts with the N-terminal pyrin domain of NLRP3 followed by oligomerization of NLRP3 with other proteins of inflammasome, apoptosis-associated speck-like protein containing a CARD (ASC) and pro-caspase-1 to form functional inflammasomes, followed by cleavage of procaspase-1 to caspase-1 and GSDMD (Xian et al., 2022; Xu et al., 2021; Lackner et al., 2025; Cabral et al., 2023). Cleaved GSDMD promotes pore formation, facilitating the release of inflammatory cytokines IL-1β and IL-18 (Xian et al., 2022; Xu et al., 2021; Lackner et al., 2025; Cabral et al., 2023). Similarly, hematopoietic interferon-inducible nuclear (HIN) domain, the C-terminus of the AIM2 protein binds to the mtDNA, which oligomerizes AIM2 and recruit ASC, followed by pore formation by cleaved GSDMD and release of IL-1β and IL-18, leading to pyroptosis (Jin et al., 2012; Hornung et al., 2009).

TLR9 is a widely studied PRR that recognizes hypomethylated CpG motifs (Newman et al., 2024; Ohto et al., 2018; Mallavia et al., 2020). It contains two DNA-binding sites and is present in the ER under resting conditions, upon activation it localizes to the endosomes (Newman et al., 2024; Ohto et al., 2018; Mallavia et al., 2020). TLR9 is predominantly expressed in immune cells, including monocytes and macrophages, and mediates the detection of mtDNA in endosomes (Newman et al., 2024; Ohto et al., 2018; Mallavia et al., 2020; Algieri et al., 2025). Immune cells take up mtDNA from damaged/dying cells via endocytosis or phagocytosis. After binding to mtDNA, it recruits myeloid differentiation primary response-88 (MyD88), which activates IRAK4, IRAK1, and TRAF6, which further activates IRF7, MAPK, and NF-κB, leading to increased expression of IL-1β and TNF-α (Algieri et al., 2025). However, in case of impaired mitophagy, mtDNA is accumulated within the endolysosomal compartment, triggering TLR9 activation and chronic inflammation (Newman et al., 2024; Ohto et al., 2018; Mallavia et al., 2020; Algieri et al., 2025; Oka et al., 2012).

mt-dsRNA is recognized by sensors like MDA5, RIG-I, PKR, TLRs in cytosol which further activates mitochondrial antiviral-signaling protein (MAVS), IRF3/NF-κB and the inflammasome pathways (Dhir et al., 2018; Chen J. et al., 2025). RIG-I specifically binds to the 5′triphosphate end of short mt-dsRNA while MDA5 recognizes the long mt-dsRNA molecule (Dhir et al., 2018; Chen J. et al., 2025; Baum et al., 2010). MDA5 interacts with mt-dsRNA and its duplex structure help in MDA5 oligomerization further leading to aggregation (Dhir et al., 2018; Chen J. et al., 2025). After activation both MDA5 and RIG-I expose their N-terminal caspase recruitment domains (CARDs) which interact with the CARD domain of MAVS present on the outer membrane of mitochondria leading to aggregation of MAVS (Dhir et al., 2018; Chen J. et al., 2025; Wu and Hur, 2015). Aggregated MAVS initiates a cascade of downstream signaling by recruiting TRAFs (such as TRAF2, TRAF3, and TRAF6) which activates two major inflammatory pathways NF-κB and IRF pathway (Wu and Hur, 2015). Recruitment of TRAF6 activates the transforming growth factor-β-activated kinase1 (TAK1) complex which phosphorylates IKK subunits leading to the release of NF-κB to the nucleus and inducing expression of pro-inflammatory cytokines (Wu and Hur, 2015; Sharma et al., 2003; Fang et al., 2017). However, the recruitment of TRAF3 to MAVS activates TBK1 and IκB kinase epsilon (IKKε), which further phosphorylates IRF3 and IRF7 leading to their dimerization and translocation to the nucleus inducing the expression of IFNs (Wu and Hur, 2015; Sharma et al., 2003; Fang et al., 2017).

Protein kinase R (PKR) interacts with mt-dsRNA, which causes PKR dimerization and autophosphorylation, following which it triggers the expression of inflammatory cytokines mediated by IKK/NF-κB and transforming growth factor-beta-activated kinase 1 (TAK1)/activator protein-1 (AP-1) signaling independent of RLR-MAVS axis (Lemaire et al., 2008; Kim et al., 2018). Furthermore, uncontrolled activation of PKR by mt-dsRNA also induces apoptosis in cells (Lemaire et al., 2008; Kim et al., 2018).

The mt-dsRNA present in the extracellular space are recognized by TLR3. After recognition, the toll-IL-1 receptor (TIR) domain of TLR3 undergoes conformational changes and phosphorylates, which induce the TIR-domain-containing adapter-inducing IFN-β (TRIF) oligomerization as well as activation of IRF3 and NF-κB to produce IFN and pro-inflammatory cytokines (Jiang et al., 2004; Kim et al., 2022). Another study has shown that mt-dsRNA can also activate immune cells through TLR8 activation which induces MyD88-mediated NF-κB activation leading to the release of TNF-α, IL-6 (Krüger et al., 2015).

Overall, these receptors recognize mtDNA and mt-dsRNA, sense them as DAMPs, and activate downstream signaling pathways, thereby perpetuating the pathogenesis of various liver diseases, as discussed in the next section.

## 5 Role of mtDNA and mtRNA in specific liver pathologies

Liver diseases are one of the leading factor of mortality globally and are responsible for approximately two million deaths every year (Devarbhavi et al., 2023; Gan et al., 2025). Acute liver diseases caused by hepatotropic virus infection, such as hepatitis B virus (HBV) and hepatitis C virus (HCV), are globally prevalent, especially in Asia, despite aggressive vaccination (Shen et al., 2026; Bai et al., 2025). Drug-induced liver injury (DILI) causes acute liver injury (ALI); despite its complicated diagnosis, DILI cases are prevalent worldwide and on rise (Björnsson and Björnsson, 2022). Similarly, the chronic liver diseases such as alcohol-associated liver disease (ALD), metabolic dysfunction-associated steatotic liver disease (MASLD), and metabolic dysfunction-associated steatohepatitis (MASH) are alarmingly increasing (Devarbhavi et al., 2023; Åberg et al., 2024; Feng et al., 2025; Younossi et al., 2025). Recent studies have found MASLD has increased rapidly and is the most prevalent chronic liver disease due to dietary habits, sedentary lifestyle, and increasing obesity (Feng et al., 2025; Younossi et al., 2025). Moreover, demographics and the population of ALD due to binge drinking have also substantially increased over the past decade (Gan et al., 2025; Åberg et al., 2024). The rapid increase in chronic liver disease cases, and their progression to end-stage liver diseases such as cirrhosis and hepatocellular carcinoma (HCC) has also substantially increased the cases of acute on chronic liver failure (ACLF) and HCC (Mauro et al., 2025; Wang et al., 2024; Kim, 2024). According to the 2024 data from NIH National Cancer Institute, liver cancer accounts for approximately one million new cases annually, which makes it the third leading cause of mortality due to cancer globally (Mauro et al., 2025). This section discusses how mitochondrial dysfunction, mtDNA and mt-dsRNA promotes the progression of these liver diseases (Table 3).

**TABLE 3:** Role of mtDNA and mt-dsRNA in the pathogenesis of liver diseases.

Liver disease	Mitochondrial DAMPs	Cause of release	Immune pathway activated	References
MASLD/MASH	mtDNA	Lipotoxicity, mitochondrial dysfunction, impaired mitophagy, oxidative stress, CMPK2-mediated mtDNA synthesis, Top1MT deficiency	TLR9, cGAS–STING, NLRP3 inflammasome, AIM2, JNK	(Pan et al., 2018; Xu et al., 2021; Mallavia et al., 2020; Gao et al., 2020; Wang Y. et al., 2022; Zhu et al., 2022; Yuzefovych et al., 2024; Yamazaki et al., 2016; Houshmand et al., 2023; Garcia-Martinez et al., 2016)
	mt-dsRNA	Mitochondrial stress and cytosolic accumulation in hepatic stellate cells	TLR3/MDA5–IRF3, Type I IFN signalling	(Travers et al., 2025; Huang et al., 2022)
ALD	mtDNA	CYP2E1/NOX4-induced oxidative stress, impaired mitophagy, ASK1/p38-dependent release of mtDNA-enriched EVs and microparticles, VDAC1-mediated leakage	TLR9, cGAS–STING, NLRP3 inflammasome	(Zhou et al., 2019; Xie et al., 2024; Ma et al., 2020; Cai et al., 2017)
	mt-dsRNA	Alcohol-induced packaging of mt-dsRNA into hepatocyte-derived exosomes	TLR3/IL-1β/IL-17A signalling	(Lee et al., 2020; Mandrekar and Mandal, 2024)
DILI	mtDNA	APAP-induced mitochondrial protein adducts, mPTP opening, Sam50–MICOS–ATAD3 axis disruption, release via EVs	TLR9, cGAS–STING, AIM2, NLRP3, JNK signalling	(Wu et al., 2019; Chen et al., 2022; Jin et al., 2026; Fromenty, 2020; Liu et al., 2022a; Marques et al., 2012; Zeng et al., 2024)
Viral Hepatitis	mtDNA	HBx- and HCV protein-mediated mitochondrial dysfunction, ROS generation, mtDNA depletion and release	cGAS–STING, TLR9, IFN signalling	(Machida et al., 2006; Selvamani et al., 2024; Jung and Kim, 2013; Bäuerle et al., 2005)
HCC	mtDNA	mtDNA mutations, copy-number depletion, oxidized mtDNA, EV-mediated transfer, DRP1 mediated release of mtDNA, HMGB induced nanotubes	TLR9, cGAS–STING, NF-κB, JAK/STAT, M2 polarization of TAMs	(Zhao et al., 2025; Chen T. H. et al., 2025; Yamada et al., 2006; Jing et al., 2024; Seo et al., 2019; Guo et al., 2022; Yang et al., 2025; Fu et al., 2025)
	mt-dsRNA	Chemotherapy dependent mitochondrial damage and release through BAX pathway	RIG-I/MAVS pathway CD8^+^ T-cell expansion	(Tian et al., 2026)

### 5.1 Metabolic dysfunction-associated steatotic liver disease (MASLD) and metabolic dysfunction-associated steatohepatitis (MASH)

Metabolic dysfunction-associated steatotic liver disease (MASLD), previously known as non-alcoholic fatty liver disease, is one of the leading causes of chronic liver disease (Devarbhavi et al., 2023; Feng et al., 2025; Younossi et al., 2025). MASLD is associated with metabolic dysregulation and is characterized by the accumulation of fat in the liver, causing hepatocyte ballooning (Devarbhavi et al., 2023; Feng et al., 2025; Younossi et al., 2025; Parthasarathy et al., 2020; Wang and Friedman, 2023). MASLD may or may not be associated with inflammation, but MASH is characterized by inflammation and can progress through varying stages of fibrosis, ultimately advancing to cirrhosis or HCC (Devarbhavi et al., 2023; Feng et al., 2025; Younossi et al., 2025; Wang et al., 2024; Parthasarathy et al., 2020; Wang and Friedman, 2023). Progression of liver injury from steatosis to MASH is a multifactorial process in which hepatocyte injury, signals from adipose tissue, and intestinal pathogens trigger inflammation (Parthasarathy et al., 2020). Damaged hepatocytes and infiltration of monocytes and neutrophils activate HSCs, leading to excessive ECM accumulation and fibrogenesis (Parthasarathy et al., 2020; Wang and Friedman, 2023; Babuta et al., 2024). However, major factors involved in the progression to MASH are *de novo* lipogenesis activation, dysregulated mitochondrial FA oxidation, and mtDNA-mediated immune response (Parthasarathy et al., 2020; Wang and Friedman, 2023).

Increase in ROS production, due to dysfunction in mitochondria, triggers apoptosis and release of pro-inflammatory cytokines (Zhang B. et al., 2022; Simões et al., 2021; Li et al., 2015). A previous study established a causal link between mitochondrial topoisomerase1 (Top1MT) and MASH progression, demonstrating that Top1MT-deficient mice exhibit mitochondrial dysfunction, accompanied by aberrant mtDNA release, thereby driving the pathogenesis of MASH (Baechler et al., 2024). Similarly, C-X-C motif chemokine receptor 3 (CXCR3) and perilipin-5 disrupt the mitochondrial homeostasis, promoting the progression of liver fibrosis (Du et al., 2017; Yin et al., 2024). *In vitro* palmitic acid (PA) treatment induces the release of mtDNA in the cytoplasm, which is recognized by NLRP3 in Kupffer cells, causing their activation and an increased secretion of IL-1β (Pan et al., 2018). Further, mtDNA released from injured hepatocytes is recognized by TLR9, which upregulates IL-33 expression along with other pro-inflammatory cytokines in macrophages (Mallavia et al., 2020; Gao et al., 2020; Shepard, 2020). In the liver of MASH patients, non-steroidal anti-inflammatory drugs activate gene-1 (NAG-1)/Growth differentiation factor-15 (GDF-15), which is downregulated induces oxidative stress, mitochondrial damage, and mtDNA release in the cytosol (Wang Y. et al., 2022). Released mtDNA is recognized by AIM2 inflammasomes, causing increased release of pro-inflammatory cytokines (Xu et al., 2021; Hornung et al., 2009).

Emerging studies show that the mitochondrial fragmentation is important for the clearance of damaged mitochondria via mitophagy and lysosome-related pathways (Uoselis et al., 2023; Wang et al., 2023). However, persistent stress and dysregulation of the mitochondrial dynamics can shift the balance towards excessive fusion, promoting the formation of megamitochondria (Wakabayashi, 2002). OPA1, an IMM protein, is responsible for mitochondrial fusion. Targeting OPA1 by disrupting its GTPase activity with the help of small molecule inhibitors such as MYLS22 and its derivative Opa1 inhibitor 0 (Opitor-0) limits the megamitochondria formation, improves mitochondria health, and markedly reduces the liver damage, and fibrosis (Ge et al., 2020; Song et al., 2007; Lee and Yoon, 2018; Yamada et al., 2022) Impaired function of hypoxia-induced gene domain family member-1a (Higd1a), an IMM protein is also associated with the progression of MASLD (Zhu et al., 2022). Studies have shown that Higd1a deficiency in hepatocytes causes free fatty acid-mediated hepatocyte apoptosis, oxidative stress, and an increase in ox-mtDNA, which further activates NLRP3 and c-Jun-N-terminal kinase (JNK) signaling pathways and, in turn, impairs FAO (Zhu et al., 2022). Similarly, mice deficient in OGG1 are susceptible to hepatic steatosis and adipogenesis, reinstating the role of ox-mtDNA in promoting the pathogenesis of MASH (Sampath et al., 2012; Yuzefovych et al., 2024). Mitochondrial enzyme activity correlates with the stage specificity of MASLD. For instance, cytidine/uridine monophosphate kinase 2 (CMPK2), an enzyme for mtDNA synthesis, is significantly upregulated in MASH patients and in a preclinical model of MASH, which activates NLRP3 inflammasome and induces pyroptosis in hepatocytes (Zhu et al., 2025). Interestingly, expression of CMPK2 is not increased in the early stages of MASLD (Zhu et al., 2025).

Recent studies suggest that mtDNA instability is a critical contributor to the cellular senescence (VanPortfliet et al., 2024). Mitochondrial dysfunction, mtDNA oxidative damage, and limited DNA repair capacity cause senescence-associated phenotypes (Wiley et al., 2016). Interestingly, a recent study showed that the mitochondrial dysfunction can be uncoupled depending on the senescence-inducing stimulus (Wiley et al., 2016). Sullivan et al., showed that TRF2 deletion-induced telomere dysfunction in mouse hepatocytes, which triggered senescence and increased Cdkn1a (p21) mRNA expression both *in vitro* and *in vivo*, without affecting mitochondrial biogenesis, respiration, metabolism, or mtDNA content (Sullivan et al., 2023). These findings indicate that mitochondrial function can remain intact in certain forms of telomere-driven senescence (Sullivan et al., 2023). These studies contrast with the established models of stress-induced senescence and suggest that the mitochondrial integrity may be preserved in specific contexts of telomere dysfunction to facilitate ongoing cellular functions (VanPortfliet et al., 2024; Wiley et al., 2016; Sullivan et al., 2023).

Epigenetic modifications of the mitochondrial genome and various mitochondrial enzymes play crucial roles in the progression of simple steatosis to MASH (Lopes A, 2020; Sharma et al., 2019). DNA (cytosine-5) methyltransferase 1 is increased in the MASH patients, suggesting a positive correlation between mtDNA methylation and disease severity (Lopes A, 2020; Sharma et al., 2019; Pirola et al., 2013). In 2016, Yamazaki *et al.* showed that the rats fed with a high-fructose diet had hypomethylated mtDNA, leading to the increased mitochondrial transcription and mtDNA content (Yamazaki et al., 2016). Further evaluation of cytosine methylation (5mC) in the mtDNA genome revealed an association between D-loop variants and lobular inflammation and advanced fibrosis stages (Hasturk et al., 2019). Further studies focused on the cytosine methylation pattern of mitochondria encoded genes such as NADH dehydrogenase 6 (*MT-ND6*), cytochrome B (*MT-CYB*), and cytochrome C oxidase (*MT-CO1*) (Hasturk et al., 2019; Pirola et al., 2021; Mposhi et al., 2023). Although *MT-ND6* mRNA was decreased in MASH patients, MT-ND6 methylation was positively associated with an increased MASLD activity score, mitochondrial defects, and peroxisome proliferation (Mposhi et al., 2023). Similarly, variation in *MT-CYB,* correlated with the mtDNA damage and was more in patients with MASH in comparison to steatosis (Pirola et al., 2021).

Additionally, to further establish the role of mtDNA in MASH, mtDNA copy number was quantified in mice fed on a high-fat diet for 20 weeks (Chen et al., 2018; Betancourt et al., 2014). A significant increase in the mtDNA as compared to nDNA was observed, which was directly proportional to the liver triglyceride content (Betancourt et al., 2014). Interestingly, studies in MASLD patients showed approximately 3.9 times increased mtDNA copy number in circulation compared to healthy controls (HC) (Houshmand et al., 2023). Plasma from mice and MASH patients showed an elevated level of ccf-mtDNA which is recognised by TLR9 and increases the inflammation and progression to fibrosis (Shepard, 2020; Garcia-Martinez et al., 2016).

Apart from the mtDNA, a recent study showed the role of mt-dsRNA in IRF3-mediated fibrosis (Travers et al., 2025). It showed TGF-β induces mt-dsRNA accumulation in the cytosol of HSCs, which further facilitates the assembly of IRF3-containing stress granules, that causes the increase in the expression of type I interferons, thereby promoting fibrosis (Travers et al., 2025). HSP60 is known to ameliorate the proteotoxicity in mitochondria, maintaining the mitochondrial homeostasis as well as in the regulation the release of mtDNA in the condition of stress (Kumari et al., 2026; Raj et al., 2026). Interestingly, Huang et al., described a novel protective mechanism of HSP60 and showed that the over-expression of HSP60 prevented the release of mt-dsRNA because of which TLR3/MDA5/IRF3 axis was not activated (Huang et al., 2022). Furthermore, they also observed decrease in mtDNA copy number and at systemic level reduced steatohepatitis (Huang et al., 2022).

Altogether, these studies highlight that mtDNA and mt-dsRNA, as important mediators which are positively associated with the pathogenesis of MASLD. In conclusion, mitochondrial dysfunction, increased mtDNA damage, mutation, release, impaired mitochondrial quality control, increased oxidative stress, mt-dsRNA release and ROS-induced inflammatory signaling play pivotal roles in the progression of MASLD to MASH.

### 5.2 Alcohol-associated liver disease (ALD)

ALD is globally prevalent and is one of the leading causes of liver transplantation (Garcia-Martinez et al., 2016; Travers et al., 2025). Individuals consuming alcohol ≥60 g daily develop steatosis, which is reversible after alcohol abstinence (Åberg et al., 2024; Crabb et al., 2020). However, continued consumption of alcohol causes the progression of ALD to fibrosis and, in some cases, to cirrhosis (Crabb et al., 2020; Wu et al., 2023). Globally, approximately 25% of liver cirrhosis is attributed to excessive alcohol consumption (Åberg et al., 2024; Crabb et al., 2020; Wu et al., 2023). Patients with heavy alcohol use develop alcohol-associated hepatitis (AH), which is mainly characterized by jaundice and approximately 90–120 days of mortality (Wu et al., 2023).

Alcohol reaches the liver via the portal vein circulation and undergoes extensive metabolism through oxidative and non-oxidative pathways (Cederbaum, 2012). The predominant route of alcohol metabolism is the oxidative pathway, which requires a NAD^+^ dependent enzyme, alcohol dehydrogenase (ALD), that converts alcohol to acetaldehyde (Cederbaum, 2012). Under physiological conditions, acetaldehyde is further oxidised into acetate by aldehyde dehydrogenase (ALDH) in mitochondria, which is further catabolized into carbon dioxide, fatty acids, and water in peripheral tissues (Cederbaum, 2012). Alcohol is also metabolized by several other enzymes by conjugation of alcohol with various endogenous molecules like fatty acid ethyl ester (FAEE), phosphotidylethanol (PEth), ethyl glucuronide (EtG), and ethylsulfate (EtS) (Cederbaum, 2012; Cederbaum et al., 2009). However, during chronic alcohol consumption, the ADH/ALDH pathway becomes saturated, leading to the accumulation of acetaldehyde and the formation of reactive aldehydes, including malondialdehyde-acetaldehyde (MAA) and 4-hydroxy-2-noneal (HNE) (Cederbaum, 2012; Cederbaum et al., 2009). These reactive metabolites can form covalent adducts with proteins, contributing to the oxidative stress and ROS generation (Cederbaum, 2012; Cederbaum et al., 2009). Increased ROS levels inhibit the downstream AMPK signaling, leading to the upregulation of sterol regulatory element binding protein 1c (SREBP-1C), downregulation of PPAR-α, and upregulation of lipogenic genes, thereby increasing fatty acid synthesis and deposition (Cederbaum et al., 2009; Attal et al., 2022; Lieber et al., 1965). This enhanced lipogenesis contributes to the alcohol-induced fatty liver (steatosis) and is one of the characteristic of early alcohol-related liver disease (Wu et al., 2023; Cederbaum et al., 2009; Attal et al., 2022; Lieber et al., 1965).

Ethanol-induced activation of CYP2E1 is considered a primary pathway responsible for the induction of oxidative stress and inflammation during ethanol exposure (Jin et al., 2013). CYP2E1 also contributes to the generation of superoxide and hydrogen peroxide during microsomal mixed-function oxidase activity (Jin et al., 2013; Abdelmegeed et al., 2017; García-Ruiz et al., 2013). Besides CYP2E1, NADPH oxidase (NOX) also contributes to the generation of ROS (Abdelmegeed et al., 2017; García-Ruiz et al., 2013; Bansal et al., 2012; Sun et al., 2017; Yu et al., 2026). NOX enzymes are the key mediators of superoxide production by transferring electrons from NADPH or NADH to molecular oxygen (García-Ruiz et al., 2013; Sun et al., 2017; Yu et al., 2026). Among the seven members of NOX family, NOX4 in particular is upregulated by the excessive alcohol consumption, leading to the dysregulation of liver mitochondria, mtDNA damage and release, and low ATP levels in the liver (García-Ruiz et al., 2013; Sun et al., 2017; Yu et al., 2026).

Mitochondrial dysfunction and lipid peroxidation act as the upstream activators of DNA-dependent protein kinase catalytic subunit (DNA-PKcs) (Zhou et al., 2019). DNA-PKcs, a regulator of mitochondrial homeostasis, is positively associated with hepatic steatosis, fibrosis, and apoptosis by promoting mitochondrial dysfunction (Zhou et al., 2019). DNA-PKcs activates p53, which further activates mitochondrial fission by increased DRP1 and reduced FUN14 domain-containing-1 (FUNDC1)-dependent mitophagy (Zhou et al., 2019). Increased mitochondrial fission and impaired mitophagy, in turn, trigger mtDNA damage, mtROS generation, and mitochondrial apoptosis, leading to the progression of ALD (García-Ruiz et al., 2013; Xie et al., 2024).

Studies have shown chronic alcohol exposure leads to the inactivation of DRP1, resulting in the formation of megamitochondria, which serves as a diagnostic tool for alcoholic liver injury and mitochondrial myopathy (Ma et al., 2023; Palma et al., 2019; Niu et al., 2026). These megamitochondria are functionally impaired, exhibiting reduced oxygen consumption and diminished phosphorylating capacities, ultimately compromising cellular energy production (Niu et al., 2026; Chedid et al., 1986). Chronic alcohol exposure triggers a cascade of mitochondrial dysfunction in the liver through multiple interconnected mechanisms (Niu et al., 2026). This mitochondrial dysfunction is closely linked to the ER stress and imbalance in the proteostasis pathway, which is impaired in AH patients (Ajoolabady et al., 2023). Studies have shown an increased protein kinase R (PKR)-like ER kinase (PERK) and activating transcription factor 4 (ATF4) signaling in AH patients, which causes impairment of mitochondrial biogenesis and respiratory function (Hao et al., 2021). Notably, the ND1 and other ND genes show reduced expression in the hepatocytes of AH patients, further compromising the mitochondrial ETC function (Xie et al., 2024). Ablation of ATF4 in hepatocytes restores mitochondrial function by regulating NRF1 and TFAM expression and ameliorates ALD (Hao et al., 2021). In ALD, the deficiency of the augmenter of liver regeneration (ALR) leads to liver fibrosis, which is associated with the portal inflammation, ductular proliferation, and activation of portal fibroblasts (Kumar et al., 2016). As observed in MASH, AH similarly involves mitochondrial dysfunction, including elevated ROS levels, reduced membrane potential, altered mitochondrial proteins and structural abnormalities which collectively triggers the non-canonical NLRP3 activation eventually leading to the hepatocyte pyroptosis (Xie et al., 2024). Further, downregulating GSDMD prevents AH-induced liver injury (Xie et al., 2024).

Circulating extracellular vesicles (EV), mainly derived from hepatocytes, are increased in AH, and their cargo comprises proteins, lipids, miRNA and mtDNA (Babuta et al., 2019; Bala et al., 2021). mtDNA in EVs is recognized by TLR9, cGAS-STING, and NLRP3 inflammasomes which influences the severity of hepatocyte damage and causes inflammation as seen in acute-on-chronic alcohol model (Ma et al., 2023; Ma et al., 2020; Cai et al., 2017). Chronic plus binge alcohol causes the release of EV, leading to steatohepatitis (Ma et al., 2020). Mechanistically, the release of mtDNA-enriched EV from hepatocytes depends on MAPK (p38) and apoptosis signal-regulating kinase 1 (ASK1), as targeting and inhibiting either ASK1 or p38 ameliorated alcohol-induced liver injury by reducing the release of mtDNA-enriched EV (Ma et al., 2020). Alcohol-induced hepatocyte injury releases the mt-dsRNA which is sensed by two convergent routes depending upon its cellular fate. In the first route, the released mt-dsRNA is packaged in exosomes, which is taken up by Kupffer cells and activates TLR3, priming NF-κB-dependent transcription of pro-IL-1β (Lee et al., 2020; Mandrekar and Mandal, 2024). Simultaneously, mt-dsRNA that escapes directly into the hepatocyte cytosol is sensed by the cytosolic sensors RIG-I and MDA-5, which engage MAVS, triggering TBK1/IRF3 and IKK/NF-κB signaling within hepatocytes (Lee et al., 2020; Mandrekar and Mandal, 2024). Both the pathways converge on NF-κB dependent priming of pro-IL-1β, which is cleaved into IL-1β by caspase-1 and stimulates T-cell differentiation into Th17 cells and IL-17A production, thus amplifying hepatic inflammation (Lee et al., 2020; Mandrekar and Mandal, 2024).

In ALD, several biomolecules that can modulate mitochondrial function are evaluated as therapeutic targets in preclinical models. For instance, IL-16 helps hepatocytes to recover from oxidised-mtDNA by arresting the cell cycle and activating DNA repair enzymes (Zhang X. et al., 2010). S-adenosylmethionine (SAM) administration inhibits the alcohol-induced mitochondrial superoxide production, mtDNA damage, and mitigates the alcohol-associated defects in the mitochondrial genome (Bailey et al., 2006). In ALD, pro-inflammatory miR-155 is significantly increased in hepatocytes and immune cells (Babuta et al., 2019). Sirtuin 1 (SIRT1) is one of the targets of miR-155–5p, which inhibits VDAC1 (Ma et al., 2025). Increase in miR-155 in ALD promotes the expression of mitochondrial VDAC1, which in turn, causes mtDNA leakage, hepatocyte senescence and inflammation (Ma et al., 2025). Dihydromyricetin (DMY) improves alcohol-induced inflammation by positively regulating miR-155-5p-SIRT1-VDAC1 (Ma et al., 2025). Quercetin, a phytochemical present in fruits and vegetables, has antioxidant and anti-inflammatory properties and is known to mitigate the ethanol-induced pyroptotic cell death by upregulating mitochondrial homeostasis via PGC-1-α, restoring the mitochondrial membrane potential, and repairing mtDNA damage (Zhao et al., 2022). Thus, mitochondrial dysfunction and mtDNA have a crucial part in the pathogenesis of ALD, and restoring mitochondrial homeostasis is an emerging therapeutic approach for mitigating ALD (García-Ruiz et al., 2013; Ma et al., 2025; Zhao et al., 2022).

### 5.3 Drug-induced liver injury

Drug-induced liver injury (DILI) causes acute liver failure and refers to the liver damage caused by pharmaceutical products or dietary supplements (Björnsson and Björnsson, 2022). Some pharmaceutical products or drugs, when taken in high doses, cause mitochondrial dysfunction by either triggering the permeabilization of the OMM, leading to severe depletion of ATP production, or by directly rupturing the mitochondrial membrane (Björnsson and Björnsson, 2022; Labbe et al., 2008).

Intrinsic or predictable DILI occurs through two distinct mechanisms: either as a result of direct toxicity in the cases of drug overdose or through the activation of immune responses (Björnsson and Björnsson, 2022). Hepatotoxicity may arise when a parent drug is bioactivated into its reactive metabolite via cytochrome P450 enzymes, rupturing the mitochondrial outer membrane, causing the activation of signaling cascade leading to apoptosis (Björnsson and Björnsson, 2022). Rupture of mitochondrial membrane also disrupts the metabolic cycles like oxidative phosphorylation and β-oxidation, resulting in microvesicular steatosis, hypoglycemia, and eventually coma or death (Björnsson and Björnsson, 2022; Labbe et al., 2008). Continuous metabolic stress or sustained exposure to the drug may evolve into steatohepatitis, fibrosis, and eventually cirrhosis and acute liver failure (ALI) (Björnsson and Björnsson, 2022; Labbe et al., 2008).

Acetaminophen-induced liver injury (AILI), is the most studied form of intrinsic DILI (Yan et al., 2018; McGill and Jaeschke, 2014). Acetaminophen (APAP) enters the liver through the portal vein circulation and the majority (∼90%) of APAP undergoes phase II metabolism and forms glucuronide and sulphate metabolites via UDP-glucouronosyl transferases (UGTs) and sulfotransferases (SULTs), which are water-soluble complexes and are excreted via urine (Yan et al., 2018; McGill and Jaeschke, 2014). Nearly 5% of APAP undergoes oxidation, forming its reactive metabolite, N-acetyl-p-benzoquinone-imine (NAPQI), via CYP2E1 (Yan et al., 2018; McGill and Jaeschke, 2014). Glutathione-S-transferases (GSTs) catalyze the detoxification of NAPQI by binding it to the reduced glutathione (GSH) and forming APAP-mercapturate (Yan et al., 2018; McGill and Jaeschke, 2014). During overdose of APAP, UGTs and SULTs get saturated and divert complete APAP metabolism towards CYP2E1 enzymes, which leads to the excessive production of NAPQI (Yan et al., 2018; McGill and Jaeschke, 2014). Subsequently, NAPQI binds to the sulfhydryl groups of glutathione and various cellular and mitochondrial proteins, causing the formation of protein adducts (Yan et al., 2018; McGill and Jaeschke, 2014). These adducts are found in circulation and are crucial for identifying DILI in patients (Yan et al., 2018; McGill and Jaeschke, 2014). The high concentration of these adducts triggers initial mitochondrial dysfunction and oxidative stress followed by mtDNA release into the cytosol (Yan et al., 2018; McGill and Jaeschke, 2014; Pessayre et al., 2012). Notably, the concentration of the adducts formed correlates with the mitochondrial protein alterations and the presence of mtDNA in the serum, both serving as vital biomarkers for mitochondrial damage (Yan et al., 2018; McGill and Jaeschke, 2014; Pessayre et al., 2012). The released mtDNA is recognized by TLR9, which in turn activates MAPK, leading to a signaling cascade and activation of ASK1, RIP1/RIP3 and MLKL (Zhang Q. et al., 2010; Wu et al., 2019; Deutsch et al., 2015). These upstream kinases activate the JNK pathway. Amplified stress, along with the formation of peroxynitrite, opens mPTP and eventually disrupts the electrochemical gradient across the IMM, leading to membrane swelling and rupture of OMM (Kon et al., 2004). Due to loss of membrane permeability, apoptotic factors like apoptosis-inducing factor (AIF), along with endonuclease G, are released in the cytoplasm and transported to the nucleus, where they cause nuclear fragmentation and manifest the progression of AILI (Susin et al., 1999; Li et al., 2001). High exposure to APAP reduces Sam50 levels, which are crucial for maintaining mitochondrial membrane integrity and mtDNA stability by interacting with the mitochondrial contact site and cristae organizing system (MICOS), a large protein complex residing in the IMM. ATAD3 is also an IMM protein that serves as an intermediary between MICOS and mtDNA, forming the Sam50-MICOS-ATAD3-mtDNA axis (Chen et al., 2022). This axis represents a critical structural and functional network that governs mitochondrial integrity, cristae organization, and mtDNA stability, thus, disruption in this axis ultimately leads to mtDNA release thereby contributing to AILI (Chen et al., 2022). Individuals susceptible to small doses of drugs can cause an increase in transaminase levels by forming conjugates between hepatic proteins and drug metabolites (Pan et al., 2025). Recent studies have demonstrated that EVs are the key mediators of intracellular communication between injured hepatocytes and immune cell activation (Babuta and Szabo, 2021). In DILI, the release of EVs by injured hepatocytes is enhanced, and these EVs selectively package contents like mtDNA, RNA, and protein including TOM20 and COX-IV (Babuta and Szabo, 2021; Jin et al., 2026). These mitochondrial constituents act as DAMPs and activate immune signaling pathways such as TLR9 and cGAS-STING, thereby amplifying the hepatic inflammatory response (Zhang Q. et al., 2010; Wu et al., 2019; Jin et al., 2026).

Several other pharmaceutical drugs can also impair mtDNA homeostasis through different mechanisms, such as inhibiting mtDNA replication, damaging mtDNA via generation of ROS, or interfering with the translation of mtDNA-encoded proteins (Fromenty, 2020). An important protein whose levels are influenced by pharmaceutical drug intake is DRP1 (Fromenty, 2020). Inhibition or deficiency of DRP1 induces mitochondrial dysfunction, resulting in elongated mitochondria, which predispose hepatocytes to LPS-induced stress (Smirnova et al., 2001; Zhang Q. et al., 2022). Due to the deficiency of DRP1, LPS-induced stress is amplified causing NLRP3 inflammasomes activation and an increase in IL-1β levels and infiltration of inflammatory macrophages (Smirnova et al., 2001; Zhang Q. et al., 2022). Stimulation with LPS elevates DRP1 levels, thereby increasing mitochondrial fission (Smirnova et al., 2001; Liu et al., 2022a). This leads to mitochondrial fragmentation and the release of mtDNA, which activates the STING pathway (Liu et al., 2022a). Furthermore, LPS also activates the unfolded protein response (UPR) in hepatocytes, promoting inositol-requiring enzyme 1α (IRE1α) mediated splicing of X-box binding protein 1 (XBP1) mRNA to generate the spliced active transcription factor XBP1s (Liu et al., 2022a). Zheng et al., showed XBP1 maintains mitochondrial integrity and function; hepatocyte-specific XBP1 deletion lead to defective clearance of damaged mitochondria, with increased ROS production and activation of NLRP3-mediated pyroptosis (Liu et al., 2022a). Furthermore, the aggravated pyropotsis causes the release of mtDNA into the extracellular milieu which when taken up by professional phagocytes activates TLR9 and cGAS-STING pathway and lead to release of IFN-γ, IL-1β, TNF-α, IL-6, and IL-10 (Liu et al., 2022a; Marques et al., 2012). The cGAS-STING pathway in the macrophages acts as an amplifier of hepatocyte injury and promotes the progression of ALI (Liu et al., 2022a; Marques et al., 2012). In AILI, leaked mtDNA is recognized by TLR9 in macrophages, which stimulates neutrophil extracellular traps (NETs) formation from the infiltrated neutrophils (Zeng et al., 2024). These NETs in turn, causes hepatocyte damage and promote release of mtDNA in cytosol which is directly recognized by AIM2 inflammasome (Zeng et al., 2024). Assembly of AIM2 recruits caspase-1 and induces IL-1β production, and pyroptosis, thereby amplifying liver inflammation (Zeng et al., 2024). The combined effects of pyroptosis of hepatocytes, infiltration of immune cells, ROS production and cytokine storm lead to hepatocellular injury, necrosis, and ultimately ALI (Pessayre et al., 2012; Fromenty, 2020; Zeng et al., 2024). mtDNA plays a critical role in DILI by serving as both a target and a trigger, activating innate immune responses that exacerbate liver inflammation (Pessayre et al., 2012; Fromenty, 2020). Thus, mtDNA integrity is central to both the initiation and amplification of DILI pathogenesis as shown in Figure 2.

**FIGURE 2:**
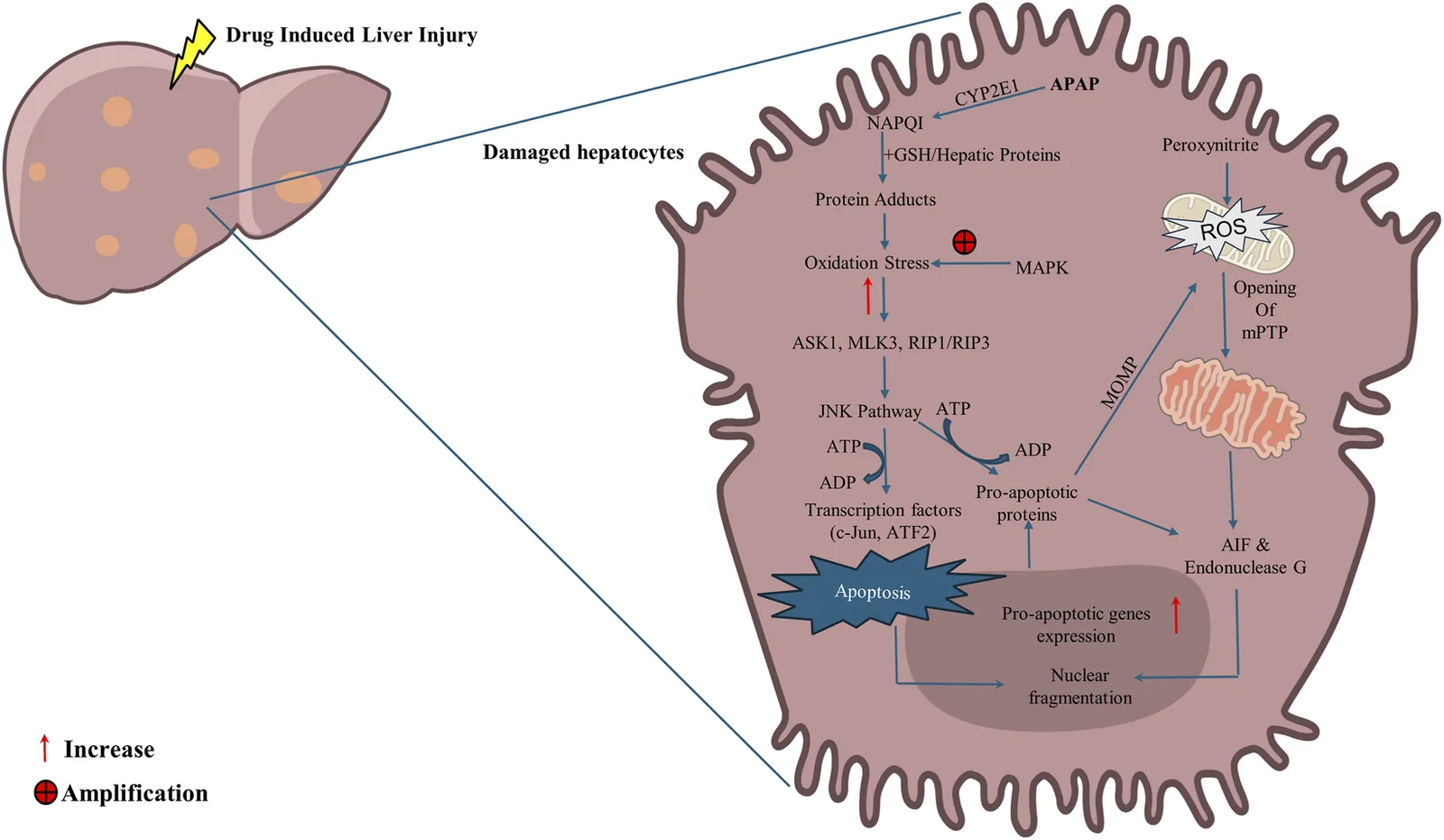
Schematic diagram showing the mitochondrial dysfunction and downstream signaling in the pathogenesis of DILI. Apoptosis signal-regulating kinase1 (ASK1), Mixed ligand kinase 3 (MLK3), Receptor interacting protein kinase (RIP1/RIP3), Mitogen activating protein kinase (MAPK), Apoptosis inducing factor (AIF).

### 5.4 Viral hepatitis

Hepatitis B virus (HBV) and hepatitis C virus (HCV) belong to the hepadnavirus and flavivirus families, respectively, and are mainly responsible for causing the viral hepatitis via targeting hepatic cells and interfering with liver metabolic activities (Guidotti and Chisari, 2006). They are mainly spread through the contact with contaminated blood or by the body fluids and can lead to persistent infection, cirrhosis, and hepatic carcinoma (Guidotti and Chisari, 2006). Both viruses can interfere with the mitochondrial activity and impair its function by decreasing the ATP production, but through different mechanisms (Guidotti and Chisari, 2006; Machida et al., 2006). HCV and HBV are associated with metabolic disorders like liver steatosis and insulin resistance (Petta et al., 2011). HCV infection causes lipid accumulation and reduced energy production, by disrupting glycolysis and β-oxidation (Machida et al., 2006; Selvamani et al., 2024). In contrast, HBV alters pyruvate metabolism by inducing pyruvate dehydrogenase kinase (PDK) and lactate dehydrogenase A (LDHA), which divert pyruvate away from energy production toward lactate formation, contributing to oxidative stress and mitochondrial damage (Machida et al., 2006; Selvamani et al., 2024).

HBV is a dsDNA virus that integrates its DNA sequences into the host genome; these sequences are transcriptionally active and produce virus-specific mRNA, like HBx protein, which may interfere with tumor suppressors and promote chronic inflammation (Guidotti and Chisari, 2006; Jung and Kim, 2013). A study reinforcing the role of HBV integration in HCC patients identified the specific integration of a fragment of the preCore/X gene into the mitochondrially encoded cytochrome c oxidase III (MT-CO3) at position ChrM9652 (Oikawa et al., 2022). The gene fragment of HBV is associated with a ‘CACCA’ microhomology sequence, suggesting a microhomology-mediated integration mechanism (Oikawa et al., 2022). HBV core protein (HBc) interferes with the host’s glutamine metabolism by causing an abnormal increase of glutamine dehydrogenase 1 (GDH1) and localizes it into the nucleus, which catalyzes the conversion of glutamate into α-ketoglutarate (α-KG) (Cheng et al., 2025). α-KG activates the transcription of HBV’s covalently closed circular DNA (cccDNA) and also directly binds to KDM4A (a histone demethylase), leading to histone demethylation on cccDNA, thus creating a favourable environment for viral replication (Cheng et al., 2025). Many studies have explored GDH1 as a therapeutic target and have used gene silencing approaches or combination therapy with existing antivirals to block GDH1 and thereby reducing viral replication (Cheng et al., 2025). HBV and HCV both manipulate mitochondrial functions, especially apoptosis and ROS production, to enhance viral survival and replication (Machida et al., 2006; Selvamani et al., 2024). The HBx binds to mPTP and prevents apoptosis in infected cells, promoting viral persistence (Jung and Kim, 2013). HBx protein, particularly its C-terminal region, promotes the formation of 8-oxoG lesions, which can lead to G:C→T:A transversions during DNA replication, leading to genome instability and mitochondrial dysfunction (Jung and Kim, 2013).

Similarly, HCV proteins like E1, E2, and NS3 also target mitochondria, which function similarly to HBx but with unique dynamics (Machida et al., 2006). The HCV E1 and E2 envelope protein induces oxidative stress by disrupting the ETC leading to the increased production of ROS, which drives chronic DNA damage, and ultimately HCC (Machida et al., 2006). NS3, acts as the primary viral protease and RNA helicase, functions alongside cofactor NS4A, cleaves MAVS which blocks the innate antiviral defense of infected cell (Kaukinen et al., 2006). This impairs the mitochondrial OXPHOS and calcium homeostasis, leading to the cellular death (Machida et al., 2006; Selvamani et al., 2024). To survive, the infected cell undergoes metabolic reprogramming and initiates the nuclear localization of hypoxia-inducible factor 1α (HIF-1α), shifting the cell towards aerobic glycolysis (Ripoli et al., 2010). Furthermore, the HIF-induced metabolic shift suppresses the host’s antiviral response (Ripoli et al., 2010). In addition to HIF-1, HIF-2 too plays a critical role in HCV infection by altering the lipid metabolism which provides a suitable environment for the proliferation of infectious viral particles (Ripoli et al., 2010). The mechanism involves both transcriptional and post-translational regulation, with evidence suggesting that the HCV proteins impair the normal degradation of HIF-1α and activate signalling pathways such as phosphotidylinositol three kinase (PI3K/Akt) and NF-κB (Ripoli et al., 2010). Among the other HCV proteins, NS4B, play a crucial role in viral RNA replication, assembly, and release (Zhao et al., 2012). It induces apoptosis via the mitochondrial death pathway, contributing to its ability to manipulate host cellular functions (Zhao et al., 2012).

The increased amount of ROS production in HCV-mediated infection causes mitochondrial dysfunction and mtDNA depletion, which is similar to the mtDNA depletion caused in HIV patients via increased oxidative stress and may also aggravate the condition to chronic hepatitis-C (Bäuerle et al., 2005). This mitochondrial dysfunction is further linked to the altered innate immune signaling where HCV mtRNA is recognized by RIG-I and MDA5, which initiates type I interferon production and is later blocked by the cleavage of MAVS by HCV’s NS3/4 A protease (Liu and Gale, 2010). In HBV infection, this pathway is instead modulated indirectly, with the virus reprogramming host cellular metabolism to blunt RIG-I/MDA5-MAVS-dependent interferon signaling rather than directly cleaving the pathway components (Zhou L. et al., 2021). Co-infection in patients with HIV/HCV shows the depleted levels of mtDNA in hepatic cells (Bäuerle et al., 2005). The most common therapy given to HCV mono-infected and HCV/HIV co-infected patients is nucleoside reverse transcriptase inhibitor (NRTIs) or nucleoside analogs (Bäuerle et al., 2005; De Mendoza et al., 2007). A combined dose of pegylated-IFN and ribavirin has been given to both the types of patients (Bäuerle et al., 2005; De Mendoza et al., 2007). This therapy significantly reduces the frequency of mtDNA mutations (De Mendoza et al., 2007). However, nucleotide analogues like lamivudine and telbivudine are shown to be associated with conditions such as toxic myopathy and peripheral neuropathy (Xu et al., 2014). They also generate mitochondrial toxicity by inhibiting DNA polymerase γ (Xu et al., 2014). Entecavir (ETV), a potent nucleoside analogue, is used to treat chronic hepatitis B infection with no mitochondrial toxicity, no altered cell proliferation, and mtDNA levels (Mazzucco et al., 2008). This contrasts with other antiviral NRTIs such as lamivudine, tenofovir, and adefovir, which show varying degrees of Pol γ inhibition and mitochondrial toxicity *in vitro* (Xu et al., 2014; Mazzucco et al., 2008). Patients suffering from HIV/HCV co-infection can also be treated via direct-acting antiviral agents (DAAs), which have significantly improved the health outcome by achieving high sustained virologic response (SVR) rates even in cirrhotic patients (Yin et al., 2022). Achieving high SVR has been associated with fewer liver-related complications (Yin et al., 2022; Elyamany et al., 2023). Another category of certain antiretroviral drugs specifically the “D drugs”, which are a group of didanosine, stavudine, and zalcitabine, inhibit the activity of DNA polymerase γ and have been shown to deplete the mtDNA levels in HIV/HCV coinfected individuals (Walker et al., 2004).

Thus, mtDNA significantly influences the course of viral hepatitis by affecting host defense mechanisms and cellular energy dynamics. Alterations in mtDNA integrity or quantity, whether due to viral activity or treatment effects, can intensify liver injury and advance disease pathology.

### 5.5 Hepatocellular carcinoma

Hepatocellular carcinoma (HCC) is one of the most aggressively growing forms of liver cancer (Mauro et al., 2025; Wang et al., 2024; Kim, 2024). Clinical representation of HCC in its early stages is often asymptomatic, making early diagnosis challenging (Mauro et al., 2025; Wang et al., 2024; Kim, 2024). Among the Asian population, main cause of HCC is HBV infection, however, in the western countries, it is HCV infection, cirrhosis, and MASH (Mauro et al., 2025; Wang et al., 2024; Kim, 2024; Dimitroulis et al., 2017). Other than HBV, HCV, and MASH, smoking, alcohol, obesity, type 2 diabetes, intake of aflatoxin, genetic predisposition, congenital abnormalities, as well as autoimmune diseases, are some of the other risk factors for HCC (Mauro et al., 2025; Wang et al., 2024; Kim, 2024; Dimitroulis et al., 2017). Additionally, an RNA virus called the hepatitis D virus, in the presence of HBV surface antigens, replicates and increases the risk of HCC (Mauro et al., 2025; Wang et al., 2024; Kim, 2024; Dimitroulis et al., 2017; Puigvehí et al., 2019).

In HCC, mtDNA accumulates mutations under oxidative stress conditions (Hsu et al., 2013; Zhao et al., 2025; Zhu et al., 2020; Xu et al., 2009; Chen TH. et al., 2025). These acquired mutations enhance mitochondrial dysfunction and ROS production, leading to tumor growth promotion (Hsu et al., 2013; Zhao et al., 2025; Zhu et al., 2020; Chen TH. et al., 2025). Similarly, various kinds of somatic mtDNA alterations, such as point mutation, insertions, mtDNA copy number changes, and deletion occurs in HCC patients (Hsu et al., 2013; Chen TH. et al., 2025; Yin et al., 2010). Screening of HCC patient samples revealed that 52% of patients have a minimum of one homo- or hetero-plasmic point mutation in the mtDNA, and 76% of those mutations were present in the D-loop, 3% in tRNA and 19% in the mRNA region (Yin et al., 2010; Yin et al., 2004; Lee et al., 2004). Among the mutations, the nonsense mutation G3842A is a specific genetic alteration in mitochondrial NADH dehydrogenase subunit I that introduces a premature stop codon, resulting in a shortened, nonfunctional protein, which impairs the functionality and assembly of complex I of mitochondria (Yin et al., 2010; Yin et al., 2004; Lee et al., 2004). Missense mutations at T6787C, G9267A, G7976A, and A11708G lead to amino acid substitutions, base-pair deletions, and insertions in 11032delA and 12418insA, which are responsible for the frameshift mutations in tRNAVal (T1659C) and tRNAAla (G5650A), altering tRNA structure and thereby promoting mitochondrial dysfunction (Yin et al., 2010; Lee et al., 2004). However, the 59% of transition mutations and mono- and dinucleotide instabilities in HCC suggest that, factors such as hepatitis B infection, liver cirrhosis, and alcohol abuse contribute to the mitochondrial damage to a greater extent (Hsu et al., 2013; Yin et al., 2010; Yin et al., 2004). Moreover, long-term alcohol consumption is linked with the accumulation of a 4,977 bp deletion in mtDNA, which is a unique feature of tumor cells and helps them adapt to a new microenvironment (Hsu et al., 2013; Yin et al., 2010; Yin et al., 2004). A decrease in mtDNA copy number leads to a point mutation in the D-loop region of the replication site, which positively correlates with tumor size, progression to liver cirrhosis, and decreased survival (Hsu et al., 2013; Chen TH. et al., 2025; Yin et al., 2010; Lee et al., 2004). Low mtDNA levels stimulate the NFκB pathway mediated inflammation which positively correlates with tumor size (Yamada et al., 2006; Thomsen et al., 2020). Interestingly, OXPHOS dysfunction is linked to mtDNA mutations that enable the tumor cells to undergo transcriptional reprogramming of genes involved in the development of invasive phenotypes associated with ECM degradation, cell motility, and cell-matrix interactions resulting in vascular invasion, metastasis, and recurrence (Barbour and Turner, 2014). Given genomic instability in HCC, studies were performed to understand how nuclear microsatellite instability (nMSI) and mitochondrial microsatellite instability (mtMSI) correlate with HCC progression (Fang et al., 2004). Results showed that mtMSI correlates with HCC development, whereas nMSI is independent of HCC (Fang et al., 2004).

Mitochondrial stress significantly upregulates amphiregulin, a ligand of epidermal growth factor receptor (EGFR), that links mitochondrial dysfunction to the malignant phenotypes in human hepatoblastoma-derived cell line (HepG2) cell (Chang et al., 2009). This upregulation increases EGFR signaling, which enhances cell survival, stimulates cell migration and promotes resistance to chemotherapeutic agents like cisplatin (Chang et al., 2009).

mtDNA plays a crucial link in HCC initiation and progression by providing a conducive tumorigenic microenvironment (Al Amir Dache and Thierry, 2023; Penta et al., 2001; Riley and Tait, 2020). For instance, in hypoxic HCC cells, high mobility group box 1 (HMGB1) expression is increased and it translocates from nucleus to cytoplasm, where it binds with TLR9 which activates p38 mediated PGC-1α activation and upregulation of mitochondrial biogenesis (Tohme et al., 2017). Furthermore, HMGB1 modulates the expression of Ras homolog family member T1 (RHOT1) and Rac family small GTPase 1 (RAC1) and HMGB1-RHOT1-RAC1 axis helps in formation of nanotubes for transfer of mitochondria to neighboring cells and promote migration and progression of HCC (Jing et al., 2024). Seo et al., showed a mitochondrial matrix protein, aldehyde dehydrogenase (ALDH2) plays a protective role in preventing HCC (Seo et al., 2019). Interestingly, ALDH2 deficiency promotes release of ox-mtDNA packaged in EVs which activates oncogenic pathways such JNK, signal transducer and activator of transcription 3 (STAT3), BCL2 and transcriptional coactivator with PDZ-binding motif (TAZ) in neighboring recipient cells (Seo et al., 2019; Cherng et al., 2021).

Recent studies have established that the ratio of mitochondrial fusion and fission proteins plays a cruical role in HCC progression and metastasis (Zhang et al., 2024; Bao et al., 2019; Lin et al., 2020; Zhang et al., 2020). For instance, protein level of mitochondrial fission protein such as mitochondrial fission regulator (MTFR)-2 and DRP1 are increased and are involved in HCC progression by promoting cell survival and proliferation (Zhang et al., 2024; Bao et al., 2019; Lin et al., 2020). However, low levels of mitochondrial fusion protein MFN1 is associated with HCC metastasis (Zhang et al., 2024; Bao et al., 2019; Lin et al., 2020; Zhang et al., 2020). Bao et al. showed that DRP1 mediated release of mtDNA in cytosol is recognized by TLR9-NF-κB signaling pathway which enhances the monocyte chemoattractant protein-1 (MCP-1) secretion which in turn promotes tumor associated macrophages (TAMs) recruitment into the tumor microenvironment (Huang et al., 2016; Bao et al., 2019). Beyond macrophage recruitment, the circulating mtDNA has also been identified as a critical regulator of macrophage polarization within the HCC microenvironment (Bao et al., 2019; Guo et al., 2022; Liu et al., 2026). Hypoxia-induced EVs are packaged with mtDNA and when taken up by macrophages activates cGAS/STING/NF-κB pathway and promotes macrophage polarization towards immunosuppressive M2 phenotype that secretes high levels of IL-10 and TGF-β (Bao et al., 2019; Guo et al., 2022; Yang et al., 2025; Fu et al., 2025). mtDNA driven M2 polarization reduces the therapeutic effect of sorafenib, a tyrosine kinase inhibitor that works as a multikinase inhibitor which slows down the growth of tumors (Yang et al., 2025). These findings highlight that prolonged mtDNA dependent inflammatory signaling favors tumor microenvironment and helps in initiation, progression and metastasis of HCC.

One of the hallmarks of the HCC is escaping the host immune response (Zhang C. et al., 2025; Chen et al., 2023). Recent studies have shown the role of RIG-I/MDA5-MAVS axis in generating anti-tumor immunity, however the role of dsRNA in tumor biology is complicated (Zhang C. et al., 2025; Chen et al., 2023). Traditionally this axis is important for antiviral immunity by activating Type I IFN response (Zhang C. et al., 2025; Chen et al., 2023). Earlier studies in HCC showed that small molecule inhibitor against TLR3, which senses dsRNA prevented cell proliferation and tumor growth (Xu et al., 2013). On the other hand, in HBV-induced HCC patients, the decreased level of dsRNA sensor PKR, is associated with poor prognosis (Chen et al., 2004). Recent studies have shown that RNA helicase DExH-box helicase 34 (DHX34) prevents immune surveillance in HCC (Zhang C. et al., 2025). Mechanistically, this study showed that DHX34 inhibition causes dsRNA accumulation followed by eliciting Type I IFN immune response via RIG-I-MAVS pathway (Zhang C. et al., 2025). Furthermore, the inhibition of DHX34 causes CD8^+^ T effector cell expansion which prevented tumor growth (Zhang C. et al., 2025). Similarly, upon chemotherapy in HCC mt-dsRNA is released in cytosol by BAX mediated pathway, which elicits an immune response through MAVS pathway (Tian et al., 2026). However, in response to accumulated mt-dsRNA tumor cells upregulate the expression of polyribonucleotide nucleotidyl transferase (PNPT1) which degrades the mt-dsRNA and prevents immune surveillance (Tian et al., 2026). Furthermore, PNPT1 inhibition prevented resistance to chemotherapy and immune checkpoint blockade (Tian et al., 2026).

Future studies deciphering the role of mt-dsRNA will shed light on its potential therapeutic role in HCC. Meanwhile, mtDNA has emerged as a potential biomarker and therapeutic interventions targeting mitochondrial dysfunction and release of mtDNA can help counter HCC.

## 6 Diagnostic and therapeutic implications of mitochondrial function and mtDNA

mtDNA positively correlates with the progression of different liver diseases. This section explores mtDNA as a potential biomarker for disease detection and for therapeutic applications.

During cellular stress, mtDNA is released in low levels into the bloodstream. This results in circulating cell-free (ccf)-mtDNA in the plasma of HCC patients, thereby increasing the sensitivity and accuracy of detection in blood samples (Yu, 2012). Studies have shown high ccf-mtDNA content is a prognostic biomarker for HCC patients receiving trans-arterial chemoembolization with traditional Chinese medicine (Yu, 2012; Zhou G. et al., 2021). In DILI, elevated mtDNA was suggested to correlate with the severity of disease and acute liver toxicity (Pessayre et al., 2012; Shi et al., 2015). In HBV patients, the release of mtDNA promotes stress, macrophage infiltration, and disease progression (Bao et al., 2019; Liu et al., 2026). Therefore, mtDNA can act as a potential biomarker for HBV patients to evaluate the risk and progression of HBV infection to cirrhosis and HCC (Bao et al., 2019; Liu et al., 2026). Variations in the D-loop have been identified to impair mtDNA replication and can be a potent biomarker for detecting MASLD and MASH progression (Paolini et al., 2023).

Studies have shown a correlation between mtDNA copy numbers and HCC progression (Yin et al., 2004; Yamada et al., 2006; He et al., 2016). The increased mtDNA copy number in circulation or elevated leuckocyte mtDNA copy number is associated with poor prognosis (Yin et al., 2004; Yamada et al., 2006). However, in solid HCC tumor, patients with low mtDNA copy number was associated with higher mortality (Yin et al., 2004; He et al., 2016). Therefore, the mtDNA copy number and its relevance in the progression of disease is context dependent (Yin et al., 2004; Yamada et al., 2006; He et al., 2016). This is evident from the fact that the alterations in mtDNA copy number are not always pathogenic and increased mtDNA copy number can exert protective effects supporting mitochondrial integrity and cellular homeostasis (Zhang et al., 2015; Mei et al., 2015; Filograna et al., 2021). Mitochondria have highly redundant and dynamic system to cope up with the mtDNA damage, to restore the functions after stress (Zhang et al., 2015; Mei et al., 2015; Filograna et al., 2021). High copy number of mtDNA can act as a sensor to mitigate mitochondrial dysfunction by inducing mitobiogenesis or repair/degradation of defective mtDNA by mitochondrial nucleases to ensure genomic stability (Zhang et al., 2015; Mei et al., 2015; Filograna et al., 2021). mtDNA copy number in circulation was measured in a study conducted with a cohort of 1,067 subjects from 18 to 93 years old (Mengel-From et al., 2014; Takahashi et al., 2018). High mtDNA copy number was positively correlated with general health (Mengel-From et al., 2014; Takahashi et al., 2018). Subjects with low mtDNA copy number associated with poor cognitive function, decreased physical strength and higher mortality (Mengel-From et al., 2014; Takahashi et al., 2018). Furthermore, studies have shown in healthy state there is a positive correlation between length of telomeres and mtDNA copy number (Gao X. et al., 2022). On the contrary, this correlation is inversed in pathological states such as chronic inflammation and HCC. For instance, in malignant HCC, telomere length is increased by ROS- protein kinase B (PKB/AKT) pathway (Ko et al., 2018). Furthermore, patients with HCC tumors with long telomere length and ROS have higher mortality rates (Ko et al., 2018).

The current therapies for mitochondrial dysfunction include antioxidant supplementation (e.g., vitamin C/E and lipoic acid), promoting mitobiogenesis, improving, ETC function, mitochondrial transplantation, and mesenchymal stem cell (MSC) therapy (Table 4) (Sinha et al., 2016). Moreover, mitochondrial transfer inhibits the formation of ROS and helps in restoring bioenergetics by incubation with purified mitochondria, microinjection, or direct transfer from donor cells like MSCs (Sinha et al., 2016; Liu et al., 2022b). MSC-based therapy is found to be efficient for the liver repair and liver regeneration (Lu et al., 2022). Studies also show that the MSCs derived from EVs cause nanotherapeutic effects by suppressing NETs formation through transferring functional to intrahepatic neutrophils (Lu et al., 2022). Recent advances in quercetin have attracted attention (Zhao et al., 2022). It protects against the alcohol-induced hepatocyte pyroptosis by downregulating mitochondrial ROS, increasing the anti-apoptotic and anti-inflammatory potential of hepatocytes, and promoting PGC-1α-regulated mitochondrial homeostasis, thereby alleviating hepatocyte pyroptosis in the human fetal hepatocyte cell line (L02 cells) (Zhao et al., 2022). Antioxidants like mitochondria-targeted ubiquinol (MitoQ), selectively target mitochondria and reduce ROS production to improve mitochondrial function, enhance endothelial function and attenuate oxidative stress (Pang et al., 2025; Dou et al., 2021). Carnitine is a cofactor required for the transport of long-chain fatty acids into mitochondria by the process of mitochondrial β-oxidation (Lim et al., 2010). Studying its effect reflected that patients administered with carnitine showed increased mtDNA copy number, which activates mitochondria helping liver to utilize fats more efficiently, ultimately contributing to the improvement of MASLD (Lim et al., 2010). Recent advances in mitochondrial RNA-based therapies can be an effective tool against mitochondrial diseases (Chernega et al., 2022). Antisense oligonucleotides (ASOs), small interfering RNAs (siRNAs), and mitochondrial microRNA can be used to therapeutically increase or decrease gene expression of defective mitochondrial RNAs and mutant mitochondrial protein (Lin et al., 2020; Chernega et al., 2022; Garcia et al., 2022; Yu et al., 2005). One such study targeted the fission factor DRP1 with ASO and found that downregulating DRP1 level not only restores mitochondrial structure, but it also rescues mitochondrial dysfunction in HCC mouse model (Lin et al., 2020; Garcia et al., 2022). Another study showed targeting DGAT2 through ASOs significantly reduced liver fat, intrahepatic TG level and blood lipid levels in obese mice (Yu et al., 2005).

**TABLE 4:** List of therapeutics to restore mitochondrial function or inhibit mtDNA release.

Therapeutic strategy	Specific Agent(s)	Mechanism of action	Effect	References
Mitochondrial Antioxidants	MitoQ, SkQ1	Targets mitochondria-derived ROS via ubiquinone moiety	Reduces oxidative stress and prevents HSC activation	(Pang et al., 2025; Dou et al., 2021)
Biogenesis Stimulators	Resveratrol, Metformin	Activates SIRT1/AMPK/PGC-1α pathway	Increases mitochondrial mass and improves fatty acid oxidation	(Cao et al., 2025)
Mitophagy Modulators	Urolithin A	Enhances PINK1/Parkin-mediated clearance of damaged mitochondria	Improves hepatocyte viability and reduces inflammation	(Gao Z. et al., 2022)
Inhibition of mtDNA Release	VBIT-4	Blocks VDAC1 oligomerization	Prevents cGAS-STING activation by mtDNA.	(Xiong et al., 2024)

In conclusion, mtDNA not only contributes to the progression of liver disease but also is responsible for its pathogenesis. Therefore, mtDNA is emerging as a potential biomarker which can be used in the early detection of disease as well as in the development of therapeutics models for MASH, ALD, DILI, and HCC. Further research is needed to focus on the precise mechanisms by which these compounds counter mitochondrial dysfunction in liver diseases.

## 7 Conclusion and future directions

In this review, we have focused on the mtDNA and mt-dsRNA, their release mechanism and recognition by cytosol DNA and RNA sensors and PRRs. Further we described the role of mtDNA and mt-dsRNA as the promoter of liver disease under lipotoxic stress, drug toxicity, viral infection or alcohol misuse. Under these stress conditions, normal homeostasis is impaired and mitochondrial dysfunction and damage promote the accumulation of mtDNA and mt-dsRNA, which are released in the cytosol due to alterations in mitochondrial membrane and formation of pore by either mPTP or VDAC oligomerization. Gasdermin-mediated pore formation and cell death pathways such as necrosis and necroptosis, also provide a route for mtDNA and mt-dsRNA escape from the mitochondria to cytosol and extracellular milieu. The released mtDNA and mt-dsRNA act as potential DAMPs, after being recognized by several PRRs such as TLR9, cGAS-STING, ZBP1, NLRP3 and AIM2 inflammasomes, which promotes the sterile inflammation in hepatocytes, HSC activation, and cause the progression of the disease to HCC. In MASLD/MASH, mtDNA accumulation and impaired mitophagy causes the release of mtDNA, further activating inflammatory pathways such as cGAS-STING and NLRP3/AIM2 inflammasome. In ALD, mtDNA and mtRNA packaged into hepatocyte-derived EVs amplifies inflammation. Similarly, in liver injuries, caused by viral hepatitis, HBx and HCV proteins disturb the mitochondrial integrity, leading to immune activation through DAMPs. Meanwhile, in HCC, mtDNA mutations, mtDNA copy number and depletion drive tumor progression and immune evasion.

One of the major drawbacks of studying the role of mtDNA in preclinical models is that the effects are often induced by high doses of APAP or fat, or by acute alcohol exposure, which does not recapitulate the spectrum of human disease. Moreover, the role of mtDNA and its impact on multi-organ damage have not been explored extensively. On the other hand, the understanding about mt-dsRNA is still not explored very well. The role of mt-dsRNA in immune surveillance or immune evasion of HCC is still at its infancy.

In totality, this comprehensive review concludes that the dysregulation of mtDNA and mt-dsRNA activates a pathogenic mechanism responsible for the progression of various liver diseases.

## Glossary

5 mC: 5-Methylcytosine
8-oxo-dG: 8-Oxo-2′-Deoxyguanosine
ADP: Adenosine Diphosphate
AIF: Apoptosis-Inducing Factor
AIM2: Absent In Melanoma 2
ALDH2: Aldehyde Dehydrogenase 2
ALI: Acute Liver Injury
AMPK: AMP-Activated Protein Kinase
APAP: N- N-acetyl-Para-Aminophenol
APAF1: Apoptotic Protease Activating Factor 1
ASC: Apoptosis-Associated Speck-Like Protein Containing a CARD
APE1: Apurinic/Apyrimidinic Endonuclease 1
APRI: Aminotransferase to Platelet Ratio Index
ASO: Antisense Oligonucleotides
ATAD3: ATPase family AAA domain-containing protein three
ATF4: Activating Transcription Factor 4
ATP: Adenosine Triphosphate
BAK: BCL2 Antagonist Killer
BAX: BCL2-Associated X Protein
BCL2: B-cell Lymphoma Protein 2
BER: Base Excision Repair
cccDNA: covalently closed circular DNA
CCL2: C-C Motif Chemokine Ligand 2
ccf-DNA: Circulating cell-free DNA
cGAS: Cyclic GMP-AMP Synthase
cGAMP: Cyclic GMP–AMP
CMPK2: Cytidine/Uridine monophosphate kinase 2
CpG: Cytosine-phosphate-Guanine
CYP2E1: Cytochrome P450 2E1
DAA: Direct-Acting Antiviral Agents
DAMPs: Damage-Associated Molecular Patterns
DILI: Drug-Induced Liver injury
D-loop: Displacement Loop
DNA: Deoxyribonucleic Acid
DRP1: Dynamin-Related Protein 1
dsDNA: Double-Stranded DNA
EGFR: Epidermal Growth Factor Receptor
ECM: Extracellular Matrix
ER: Endoplasmic Reticulum
ERRα: Estrogen-related Receptor α
ETC: Electron Transport Chain
ETV: Entecavir
FAO: Fatty Acid Oxidation
FADH2: Flavin Adenine Dinucleotide (Reduced Form)
FEN1: Flap Endonuclease 1
FIS1: mitochondrial Fission 1 protein
GDF-15: Growth Differentiation Factor 15
GDH1: Glutamine Dehydrogenase 1
GSDMD: Gasdermin D
GTPase: Guanosine Triphosphatase
HBV: Hepatitis B virus
HCC: Hepatocellular Carcinoma
HCV: Hepatitis C Virus
HIF-1α: Hypoxia Inducible Factor 1α
HIN Domain: Hematopoietic Interferon-inducible Nuclear Domain
HIV: Human Immunodeficiency Virus
HK2: Hexokinase 2
HSCs: Hepatic Stellate Cells
HSP: H strand promoter
IFI-16: Interferon Gamma-Inducible Protein 16
IFN: Interferon
IFN-γ: Interferon gamma
IKK: IκB Kinase
IL-18: Interleukin 18
IL-1β: Interleukin 1 Beta
IMM: Inner Mitochondrial Membrane
IRF: Interferon Regulatory Factor
ISG: Interferon-Stimulated Genes
JNK: c-Jun N-terminal Kinase
KCs: Kupffer cells
LDHA: Lactate Dehydrogenase A
LRR: Leucine-Rich Repeat
LPS: Lipopolysaccharide
LSECs: Liver Sinusoidal Endothelial Cells
LSP: L strand promoter
MASLD: Metabolic Dysfunction-Associated Steatotic Liver Disease
MAPKs: Mitogen-Activated Protein Kinases
MAVS: Mitochondrial Antiviral Signaling Protein
MDA5: Melanoma Differentiation-Associated Gene 5
Mdivi1: Mitochondrial Division Inhibitor 1
MDVs: Mitochondria-Derived Vesicles
MFF: Mitochondrial Fission Factor
MFN1: Mitofusin 1
MFN2: Mitofusin 2
MICOS: Mitochondrial Contact Site and Cristae Organizing System
MiD49: Mitochondrial Dynamics protein of 49 kDa
MIM: Mitochondrial Inner Membrane
MIRO: Mitochondrial Rho GTPase
MLKL: Mixed Lineage Kinase Domain-Like Protein
MOM: Mitochondrial Outer Membrane
MOMP: Mitochondrial Outer Membrane Permeabilization
mPTP: Mitochondrial Permeability Transition Pore
mtDAMPs: Mitochondrial Damage-Associated Molecular Patterns
mtRNA: Mitochondrial RNA
MT-CO1: Mitochondrially Encoded Cytochrome C Oxidase I
MT-CO3: Mitochondrially Encoded Cytochrome C Oxidase III
MT-CYB: Mitochondrially Encoded Cytochrome B
mtDNA: Mitochondrial DNA
mtSSB: Mitochondrial Single Stranded Binding protein
MTERF1: Mitochondrial Termination Factor 1
mtMSI: Mitochondrial Microsatellite Instability
MT-ND6: Mitochondrially Encoded NADH Dehydrogenase 6
MyD88: Myeloid Differentiation Primary Response 88
NADH: Nicotinamide Adenine Dinucleotide (Reduced Form)
NADPH: Nicotinamide Adenine Dinucleotide Phosphate
NAFLD: Non-Alcoholic Fatty Liver Disease
NAG-1: NSAID-Activated Gene 1
NAPQI: N-Acetyl-P-Benzoquinone-Imine
NASH: Non-Alcoholic Steatohepatitis
nDNA: Nuclear DNA
NETs: Neutrophil Extracellular Traps
NF-κB: Nuclear Factor Kappa-light-chain-enhancer of Activated B Cells
NLRP3: NOD-Like Receptor Protein 3
nMSI: Nuclear Microsatellite Instability
NOXs: NADPH Oxidases
NRF: Nuclear Respiratory Factor
NRTIs: Nucleoside Reverse Transcriptase Inhibitor
NSAID: Non-Steroidal Anti-Inflammatory Drug
OGG1: 8-Oxoguanine DNA Glycosylase 1
OMM: Outer Mitochondrial Membrane
OPA1: Optic Atrophy Protein 1
OXPHOS: Oxidative Phosphorylation
PDK: Pyruvate Dehydrogenase Kinase
PERK: Protein Kinase R (PKR)-like Endoplasmic Reticulum Kinase
PGC: Proliferator-Activated Receptor Coactivators
PI3K: Phosphatidylinositol Three Kinase
PINK1: PTEN-Induced Kinase 1
PMF: Proton Motive Force
POLγ: DNA Polymerase Gamma
POLRMT: RNA polymerase, Mitochondrial
PRRs: Pattern Recognition Receptors
PTM: Post Translational Modifications
PYD: Pyrin Domain
RAB5: Ras-Associated Binding Protein 5
RAB7: Ras-Associated Binding Protein 7
RIG-1: Retinoic Acid-Inducible Gene I
RITOLS: RNA Incorporation Throughout the Lagging Strand
RIPK1: Receptor-Interacting Protein Kinase 1
RIPK3: Receptor-Interacting Protein Kinase 3
ROS: Reactive Oxygen Species
SIRT4: Sirtuin 4
siRNA: Small Interfering RNAs
SNP: Single Nucleotide Polymorphism
SNX9: Sorting Nexin 9
STAT3: Signal Transducer and Activator of Transcription 3
STING: Stimulator of Interferon Genes
SVR: Sustained Virological Response
TAM: Tumor-Associated Macrophages
TAZ: Transcriptional Coactivator with PDZ-Binding Motif
TBK1: TANK-binding Kinase 1
TCA: Tricarboxylic Acid
TEFM: Transcription Elongation Factor, Mitochondrial
TF2BM: Transcription Factor 2B, Mitochondrial
TFAM: Mitochondrial Transcription Factor A
TLR: Toll-Like Receptor
TLR9: Toll-Like Receptor 9
TNF-α: Tumor Necrosis Factor Alpha
Top1MT: Mitochondrial Topoisomerase 1
TRAF6: TNF Receptor-Associated Factor 6
UPR: Unfolded Protein Response
VDAC: Voltage-Dependent Anion Channel
VDIMs: VDAC1-Derived Inner Membrane Vesicles
VPS35: Vacuolar Protein Sorting 35
XBP1: X-box Binding Protein 1
ZBP1: Z-DNA Binding Protein 1
α-KG: α-ketoglutarate
